# Robust and Thermally Stable Silicone Aerogels with Hyperconnected Network via Kinetically Optimized Hyperbranched Silane Precursors

**DOI:** 10.1002/advs.75069

**Published:** 2026-03-31

**Authors:** Aoqing Yan, Guixiang Li, Yaolan Li, Yi Luo, Bin Liu, Zhe Su, Hao Tian, Wei Shi, Bo Niu, Donghui Long

**Affiliations:** ^1^ Key Laboratory of Specially Functional Polymeric Materials and Related Technology (Ministry of Education) School of Chemical Engineering East China University of Science and Technology Shanghai China; ^2^ Suzhou Laboratory Suzhou Jiangsu China

**Keywords:** extreme environment applications, hyperbranched, kinetic optimized, mechanical strength and thermal stability, silicone aerogels

## Abstract

The development of high‐performance silicone aerogels is often limited by a trade‐off between mechanical strength and thermal stability. Distinct from the stochastic co‐polymerization of traditional networks that yield heterogeneous structures, we present a kinetic‐control strategy enabling the precise construction of a robust and thermally stable silicone aerogel (PSA) with a hyperconnected network. This is achieved by co‐condensing novel hyperbranched siloxane‐amino/epoxy (SAE) nodes with linear polymethylhydrosiloxane (PMS). The hyperbranched SAE is formed through temperature‐regulated polycondensation exploiting the kinetic disparity between methoxy‐ and ethoxy‐silane precursors, which is hypothesized to direct the reaction toward intermolecular crosslinking while suppressing intramolecular cyclization. This ensures uniform incorporation of flexible segments and abundant reactive sites. Subsequent co‐condensation yields a highly crosslinked framework with embedded flexible segments, providing superior mechanical strength and thermal stability with minimal organic content. The optimized PSA exhibits high compressive strength (7.1 MPa), low density (0.32 g·cm^−^^3^), low thermal conductivity (0.032 W·m^−^^1^·K^−^^1^), and high char yield (68% at 800°C). The quartz‐fiber composite (PSC) achieves high tensile strength (28.1 MPa) and excellent thermal‐insulation and ablation resistance up to 1000°C. This reactivity‐programmed assembly establishes a new paradigm for decoupling strength and thermal stability in hybrid aerogels.

## Graphical abstract

Reactivity‐Programmed Assembly: A kinetic‐control strategy is reported for constructing hyperconnected silicone aerogels with a robust “thick‐neck” architecture. By exploiting the reactivity disparity of precursors, flexible segments are uniformly embedded within a rigid skeleton. This multiscale design effectively decouples mechanical strength from thermal stability, yielding advanced materials with superior insulation and ablation resistance.

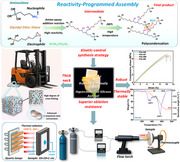

## 1 Introduction

High‐performance insulation materials that exhibit a superior combination of mechanical strength and thermal stability are critically needed for extreme environments such as aerospace applications [1, 2, 3, 4, 5]. Historically, silica aerogels have been prime candidates due to their ultralow density, excellent high‐temperature resistance, and outstanding thermal insulation properties [6, 7, 8]. However, their practical application has been substantially limited by an inherent brittleness, which originates from the fragile neck connections between aggregated nanoparticles [9, 10, 11]. An effective strategy to improve their toughness is to incorporate organic components, forming hybrid silicone aerogels. This can be achieved by using silane precursors with functional organic side chains [12, 13] or by co‐polymerizing with epoxy resins [14, 15, 16] or isocyanate [17, 18, 19] networks. Nevertheless, these strategies generally introduce a challenging performance trade‐off: significant toughening typically requires a high organic content, which inevitably compromises the aerogel's thermal stability and increases its density. More fundamentally, traditional co‐polymerization methods lack precise kinetic control. Because monomers with varying reactivities are often mixed indiscriminately, the uncoordinated and chaotic sol‐gel transitions typically lead to structural imprecision, manifesting as random phase separation, non‐uniform particle aggregation, and an abundance of unreacted dangling chains [20, 21]. These heterogeneous, defective network structures inherently limit the aerogel's ultimate mechanical strength and thermal stability.

Recent efforts have focused on silicone aerogels with organic‐inorganic double networks. For example, mechanical properties can be enhanced by the free‐radical polymerization of olefinic monomers, generating polymer chains that are grafted onto the Si‐O‐Si structure [9]. While silicone aerogels with enhanced mechanical strength have been reported using this approach with polystyrene or poly(butyl acrylate) [22, 23], it is not without drawbacks. Most of these enhancement treatments are performed post‐gelation, involving cumbersome procedures and increased costs. Furthermore, despite the polymer chains being “anchored” to the silica surface, a distinct organic‐inorganic interface persists. This interface can become a point of stress concentration and preferential failure under high temperature or stress, thereby limiting the material's ultimate thermal stability and mechanical strength. An alternative approach involves pre‐synthesizing a hybrid organic‐inorganic intermediate, which is then used in a subsequent sol‐gel process [10, 24]. For instance, Ren et al. prepared a multifunctional cage‐like silsesquioxane nano‐crosslinker and co‐condensed it with methyltriethoxysilane to prepare a hybrid silica aerogel [8]. However, the “rigid‐flexible” design, aimed at enhancing toughness, yielded a low‐modulus elastomer. Such materials undergo significant deformation under low stress and are thus unsuitable for load‐bearing applications in extreme environments.

Herein, to explicitly circumvent the aforementioned structural imprecisions, we report a kinetic‐control strategy for the precise construction of a robust and thermally stable silicone aerogel (PSA) with a hyperconnected network. The synthesis of the key component, a novel hyperbranched siloxane‐amino/epoxy (SAE) node, is critically governed by a temperature‐regulated polycondensation that skillfully leverages the kinetic disparity between methoxy‐ and ethoxy‐silane precursors. This approach is hypothesized to enforce a sequential polymerization pathway, wherein fast‐reacting methoxy groups initially form oligomeric cores that are subsequently bridged by slower ethoxy groups. Such kinetic regulation strategically biases the reaction toward intermolecular crosslinking while suppressing wasteful intramolecular cyclization, thereby achieving the uniform incorporation of flexible carbon‐chain segments and abundant reactive sites. Finally, the co‐condensation of these optimized SAE nodes with linear polymethylhydrosiloxane (PMS) generates a highly crosslinked PSA framework. This resulting architecture embeds the flexible segments directly into the silica backbone, yielding superior mechanical strength and thermal stability with minimal organic content. Furthermore, its quartz fiber mat reinforced composite (PSC) further delivers superior tensile strength as well as excellent high‐temperature insulation and ablation resistance up to 1000°C. Together, these results highlight not only the outstanding performance of PSA and PSC but also establish a clear kinetic paradigm for the rational design of advanced hybrid aerogels.

## 2 Results and Discussion

### 2.1 Synthesis of Hyperbranched Siloxane–Amino/Epoxy Resin (SAE)

**FIGURE 1:**
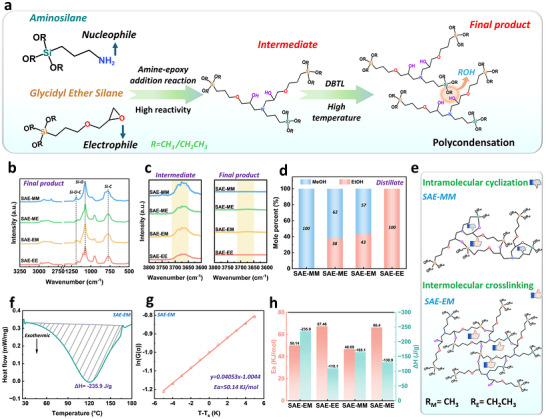
Synthesis Mechanism and Chemical Evolution of SAEs. (a) Schematic of the reaction mechanism for SAE. (b) FT‐IR spectra of the final product. c FT‐IR spectra (hydroxyl region) comparing intermediates and final products. (d) Molar percentages of methanol and ethanol in the distillates collected during the syntheses of various SAE. (e) Schematic illustration of the dominant polymerization mechanisms for SAE‐EM and SAE‐MM. (f) Representative non‐isothermal DSC curve of SAE‐EM. (g) Representative Horowitz‐Metzger (HM) plot for SAE‐EM, showing the linear fit used to calculate the activation energy (*E_a_*). (h) Summary and comparison of the key thermodynamic (Δ*H*) and kinetic (*E_a_*) parameters for all four SAE precursors, quantitatively proving the success of the kinetic optimization strategy.

The hyperbranched SAE was synthesized through a temperature‐controlled, sequential amine/epoxy addition and polycondensation process. As illustrated in Figure 1a, the process initiated with the facile nucleophilic addition of an amino silane to an electrophilic glycidyl ether silane. This crucial step generated reactive hydroxyl groups in situ. Subsequently, upon heating in the presence of a DBTL catalyst, these newly formed hydroxyls drove the polycondensation of alkoxy groups, releasing alcohol (ROH) as a byproduct to form a cross‐linked, hyperbranched topology. This efficient, sequential chemistry enabled the rational design of a unique molecular architecture distinguished by long flexible chains and a high density of reactive functionalities.

To further investigate the reaction mechanism and the effect of different alkoxy group combinations on the reaction process, a comprehensive chemical structure analysis was conducted on the reaction intermediates, the final products, and the byproducts collected via fractional condensation (Figure 1). As shown in the Fourier‐transform infrared (FT‐IR) spectra of the intermediates (Figure 1c; Figure S2), all samples exhibit a distinct absorption peak in the 3650−3700 cm^−1^ region [14, 25]. This peak is characteristic of the O─H stretching vibration in hydroxyl groups, confirming that the amine‐epoxy addition reaction occurred in all samples after Step 1, leading to the in‐situ formation of reactive hydroxyl groups. Furthermore, to accurately track the reaction progress, the unreacted raw material mixtures were supplemented for longitudinal comparison (Figure S2). The raw materials exhibit a perfectly flat baseline in the 3650−3700 cm^−1^ region. Following Step 1, as magnified in Figure 1c, a distinct newly generated hydroxyl (‐OH) peak emerges in all intermediates, serving as a highly reliable and direct indicator of the epoxy ring‐opening extent. Notably, the intensity of this newly generated hydroxyl peak is significantly stronger in the intermediates of SAE‐EM and SAE‐MM compared to the other two samples. This indicates that the amine‐epoxy addition reaction is more complete in these two variants, consequently generating a higher concentration of reactive hydroxyl groups. The enhanced reactivity is attributed to the epoxy silane bearing methoxy groups, which exhibits a higher activity in addition reaction than its ethoxy‐bearing counterpart, a difference attributed to two primary factors: first, the lower steric hindrance of the methoxy group relative to the ethoxy group, and second, the slightly stronger electron‐withdrawing effect of the methoxy group, which increases the partial positive charge on the epoxide ring and renders it more susceptible to nucleophilic attack by the amine. These additional hydroxyls, in turn, provided more available sites for the subsequent polycondensation reaction in Step 2.

Subsequently, the final products were analyzed using FT‐IR spectroscopy, with the results presented in Figure 1b. A clear observation is the complete disappearance of the hydroxyl infrared absorption peak in the 3650–3700 cm^−1^ region, which confirms the full consumption of the reactive hydroxyl groups during the Step 2 polycondensation. The physical transformation of the materials is illustrated in Figure S3. After Step 1, the reaction intermediate remained a clear, transparent liquid with a low viscosity (∼1.86 mPa·s), similar to the raw materials. However, following Step 2, the product transformed into a yellow, highly viscous liquid. As shown in Figure S4, the final viscosities diverge significantly: SAE‐MM (2.05 Pa·s) and SAE‐EM (4.76 Pa·s) are several times more viscous than SAE‐ME (0.65 Pa·s) and SAE‐EE (0.46 Pa·s). This disparity arises partly because SAE‐MM and SAE‐EM, having undergone a more complete initial reaction, possessed a higher concentration of reactive sites for polycondensation.

A noteworthy anomaly, however, is the significantly higher viscosity of SAE‐EM compared to SAE‐MM. This phenomenon is rooted in the differential reactivity of the alkoxy groups, which dictates the balance between two competing pathways during polymerization: intermolecular crosslinking and intramolecular cyclization (Figure 1e). In the homogeneous SAE‐MM system, all condensable sites are highly reactive methoxy groups. This leads to an extremely rapid, uncontrolled reaction at 140 °C that promotes unproductive intramolecular cyclization. This process results in more compact, globular molecules with less chain entanglement, which manifests macroscopically as a lower viscosity. In contrast, the heterogeneous SAE‐EM system contains both highly reactive methoxy groups and less reactive ethoxy groups. This reactivity differential is hypothesized to promote a more controlled, kinetically differentiated polymerization process. Given the significantly higher reactivity of the methoxy groups, it is highly plausible that they preferentially condense in the early stages to form branched oligomeric intermediates. As the reaction progresses, the remaining less reactive ethoxy groups likely facilitate efficient intermolecular crosslinking between these growing intermediates. This growth mechanism suppresses premature cyclization and favors intermolecular linking, resulting in a polymer with a higher average molecular weight, a more entangled network, and thus a markedly superior viscosity.

To quantitatively validate this competitive mechanism and the resulting differences in crosslinking efficiency, a stoichiometric back‐calculation was performed based on the total distillate mass and precise MeOH/EtOH molar ratios (Table S1). Since each molecule of evolved alcohol corresponds to the consumption of one hydroxyl group forming a Si‐O‐C linkage, the absolute moles of evolved alcohols directly reflect the extent of the dealcoholization polycondensation. The calculation reveals a compelling counter‐intuitive phenomenon: SAE‐MM exhibits the highest reaction extent (0.932 mol, consuming 93.2% of the theoretical hydroxyls), yet its apparent viscosity is significantly lower than that of SAE‐EM. This stoichiometric anomaly (higher conversion but lower relative viscosity) perfectly corroborates that a vast majority of the highly reactive methoxy sites in SAE‐MM were indeed consumed by wasteful intramolecular cyclization, forming compact micro‐gels rather than contributing to macroscopic chain entanglement. Conversely, the slightly lower overall reaction extent (0.869 mol) but substantially higher viscosity of SAE‐EM confirms that the kinetic disparity successfully directed the reaction toward massive intermolecular crosslinking.

To further elucidate the reaction mechanism of the SAEs, the volatile byproducts generated during the polymerization process were collected and characterized. The FT‐IR spectra (Figure S5a) of all distillates exhibit a broad absorption peak around 3300 cm^−1^, which is characteristic of the hydroxyl (‐OH) group in alcohols. The distillate from SAE‐MM displays a spectral profile that perfectly matches that of standard methanol, while the distillate from SAE‐EE shows characteristic absorptions for ethanol [26]. The spectra for SAE‐ME and SAE‐EM contain the characteristic peaks of both methanol and ethanol, confirming that these byproducts were alcohol mixtures [26, 27]. The ^1^H NMR spectra (Figure S5b) provide more definitive evidence for these conclusions, with each distillate exhibiting the expected signals for methanol, ethanol, or a mixture of them [28, 29]. The relative molar contents of the distillates were analyzed using gas chromatography (GC), with the results presented in Figure 1d. In agreement with the preceding spectroscopic discussion, the distillates from SAE‐MM and SAE‐EE were confirmed to be pure methanol and pure ethanol, respectively. In the distillate from the SAE‐EM synthesis, the mole percent of methanol reaches 57%, indicating that most of the condensation reactions occurred with the highly reactive methoxy groups. However, a counter‐intuitive result is observed for SAE‐ME: although its precursor formulation had a higher overall ethoxy content, the resulting distillate contains 62% methanol by mole. This outcome is attributed to the less complete epoxy ring opening during Step 1 for this variant, which generated a lower total concentration of reactive hydroxyl sites. During the subsequent polycondensation, it is highly likely that the more reactive methoxy groups preferentially consumed this limited supply of hydroxyls, consequently leaving an insufficient number of reactive sites for the less reactive ethoxy groups to participate fully. Ultimately, all synthesized samples feature an alkoxy‐rich hyperbranched architecture. This structure provides an abundance of reactive functional groups for the subsequent synthesis of silicon aerogels (Figure S6). Collectively, these analytical results demonstrate that the choice of precursor alkoxy groups profoundly influences the kinetic pathways of polymerization. This, in turn, ultimately governs the degree of hyperbranching and the final properties of the product.

To quantitatively elucidate the kinetic origin of the structural evolution, the polymerization thermodynamics and kinetics of the four precursors were analyzed by differential scanning calorimetry (DSC) (Figure 1f–h; Figure S7). As shown in Figure 1h, the SAE‐EM precursor exhibits the largest exothermic enthalpy change (ΔH = −235.9 J·g^−^^1^), which is markedly higher than those of SAE‐MM (−165.1 J·g^−^^1^), SAE‐ME (−130.9 J·g^−^^1^), and SAE‐EE (−110.1 J·g^−^^1^). This thermodynamic evidence confirms that the SAE‐EM system achieves a substantially higher degree of polymerization and crosslinking. Furthermore, the apparent activation energy (*E_a_*) of the curing reaction, determined using the Horowitz–Metzger method, reveals a clear kinetic hierarchy [30]. SAE‐MM exhibits the lowest *E_a_* (46.69 kJ·mol^−^^1^), consistent with the high reactivity of methoxy groups that induces rapid but disordered intramolecular cyclization. In contrast, SAE‐EE shows the highest *E_a_* (67.46 kJ·mol^−^^1^), reflecting the sluggish condensation of ethoxy groups with a higher energy barrier. The SAE‐ME variant displays a similarly high *E_a_* (66.40 kJ·mol^−^^1^), indicating that its kinetic pathway is nearly as unfavorable as that of the pure ethoxy system. Strikingly, the optimized SAE‐EM precursor exhibits an intermediate yet ideal *E_a_* (50.14 kJ·mol^−^^1^). The kinetic disparity between methoxy and ethoxy groups is the decisive factor in regulating the network topology. In the SAE‐MM system, the ubiquitous high reactivity of methoxy groups drives rapid intramolecular cyclization, causing the premature consumption of active hydroxyl groups within discrete molecules. This self‐looping prevents effective chain extension, resulting in low‐molecular‐weight structures where flexible segments are trapped in local clusters. In contrast, the SAE‐EM system follows a sequential polymerization pathway: the highly reactive methoxy groups preferentially react in the early stage to assemble initial oligomeric cores, while the less reactive ethoxy groups are reserved for the subsequent stage to bridge these oligomers together. This sequential mechanism effectively suppresses unproductive cyclization and promotes the formation of a hyperbranched network with a significantly higher degree of polymerization and uniformly incorporated flexible segments.

### 2.2 Design and Structure of PSA

**FIGURE 2:**
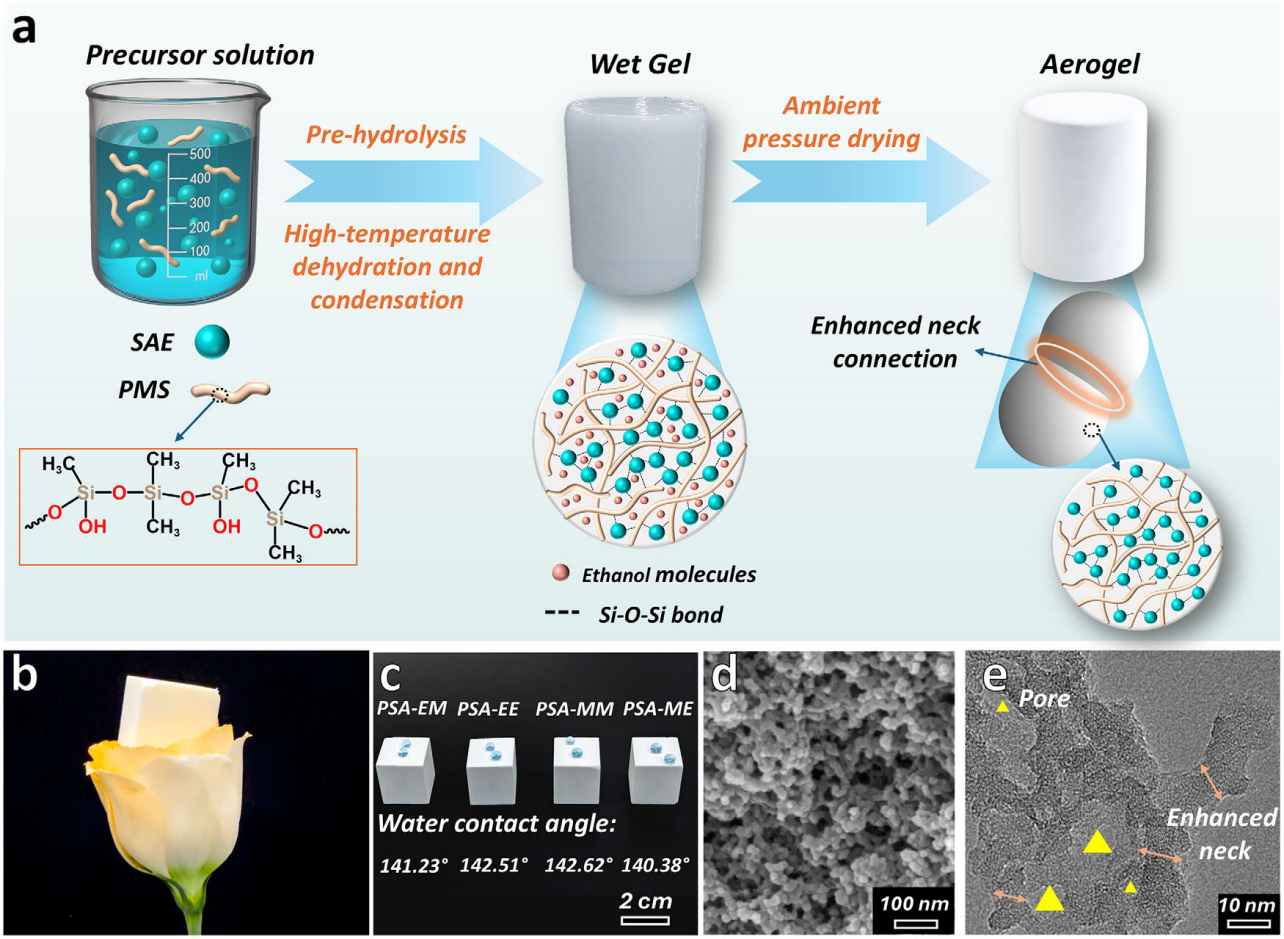
Design and architecture. (a) Schematic depicting the fabrication process of PSA. (b) A photograph showing PSA‐EM resting on a flower. (c) Digital photograph highlighting the hydrophobicity of PSAs. d, e SEM (d) and TEM (e) images of PSA‐EM.

The PSA were synthesized via the sol–gel polymerization of PMS and SAE in ethanol, followed by drying at ambient pressure (Figure 2a). In this process, PMS and SAE were dissolved in ethanol, followed by the addition of a stoichiometric amount of water. The mixture then underwent a pre‐hydrolysis step at 40°C for 2 h, yielding a clear solution. The amine groups within the SAE structure provide a self‐catalytic function for hydrolysis, which obviated the need for an external catalyst at this stage. Notably, the pre‐hydrolyzed PSA‐MM solution became turbid after aging for 6 h at room temperature (25°C), whereas all other sample solutions remained clear (Figure S8). This instability is a direct consequence of the higher reactivity of methoxy groups compared to ethoxy groups, which allowed for substantial condensation and subsequent phase separation even without a catalyst or heat [31].

After catalyst addition, the precursor solution was transferred to a sealed container and cured at 60°C to form a continuous three‐dimensional network. The resulting gel was then aged stepwise at 100°C and 150°C to strengthen its skeletal framework. The abundance of crosslinking sites (Si‐OH) on both SAE and PMS precursors results in PSAs with a high degree of crosslinking and robust inter‐particle necking, ultimately developing a hyperconnected network with superior mechanical strength. Specifically, this hyperconnectivity is distinguished by a high degree of siloxane condensation (predominantly T^3^ units) and thickened, continuous interparticle necks, which minimize condensation defects and enable efficient load transfer across the network. Furthermore, the long carbon chains inherent to the SAE structure enhance the toughness of the PSA network, mitigating the intrinsic brittleness of conventional silica aerogels. The resulting PSAs exhibit densities in the range of 0.32−0.35 g·cm^−3^ and a volumetric shrinkage of less than 7% (Table S3). Its comparatively low density enables it to be placed on a flower without causing it to bend or fall (Figure 2b). Their robust network structure withstands the high capillary forces during solvent evaporation, enabling direct ambient pressure drying. This process circumvents the need for costly procedures like solvent exchange and supercritical drying, offering significant economic and environmental advantages. The PSAs were fabricated into large, easily machined monoliths, highlighting their suitability for diverse applications (Figure S9). As shown in Figure 2c, all samples display excellent hydrophobicity, with water contact angles exceeding 140°, effectively resolving the intrinsic issue of moisture absorption in conventional silica aerogels.

SEM and TEM imaging were employed to investigate the microstructure of PSAs, which reveal that the PSAs consist of aggregated nanoparticles forming a network with robust, thick inter‐particle necks, which contrasts sharply with the fragile “pearl‐necklace” structure of conventional silica aerogels (Figure 2d,e; Figure S10) [10, 32]. Elemental mapping further confirms a uniform distribution of Si, O, and C, with trace amounts of N (Figure S11). The porous characteristics of the PSAs were characterized by nitrogen adsorption‐desorption isotherms (Figure S12). The isotherms exhibit Type IV behavior with a distinct hysteresis loop in the P/P_0_ range of 0.8–0.9, which is indicative of a mesoporous structure [9]. The aerogels have high BET specific surface areas of 435−537 m^2^·g^−1^, large total pore volumes of 1.42–1.59 cm^3^·g^−1^, and average pore diameters of 9.4–12.1 nm, as summarized in Table S3.

Chemical structure analyses were performed to confirm the composition and network integrity of the PSAs (Figures S13 and S14). Structural analyses via XRD and FT‐IR spectroscopy confirm that all PSAs possess a primary backbone of an amorphous Si‐O‐Si network, with successful incorporation of organic Si‐CH_3_ groups from the PMS precursor [33, 34, 35]. Furthermore, the FT‐IR analysis reveals that residual epoxy groups from the SAE precursor underwent additional crosslinking reactions during the high‐temperature aging step, which contributes to the enhanced mechanical properties of the final aerogels. The detailed mechanism for this reaction is discussed in previous work [14]. Investigation of the silicon chemical environments by ^2^ ^9^Si NMR spectroscopy (Figure S14c,d) reveals that the PSA‐EM network consists predominantly of fully condensed, triply bridged (T^3^) silicon units, indicating a highly crosslinked and structurally integral framework. In contrast, the other PSA variants (PSA‐MM, PSA‐ME, and PSA‐EE) exhibit a considerable proportion of less‐condensed T^2^ units, signifying a higher concentration of structural defects [8, 36, 37]. This finding confirms that the unique precursor chemistry of PSA‐EM leads to a more perfectly formed network structure.

The surface chemical composition of the PSAs was further analyzed by X‐ray photoelectron spectroscopy (XPS). Survey scans (Figure S14b) confirm that PSAs consist of Si, O, and C, along with trace amounts of N, consistent with the precursor composition. The high‐resolution C 1s spectrum (Figure S14e) can be deconvoluted into three peaks assigned to C—Si, C—C, and C—O bonds, respectively, clearly reflecting the organic functional groups within the aerogel [7]. Crucially, analysis of the high‐resolution Si 2p spectrum (Figure S14f) reveals the chemical environment of the silicon centers. The spectrum is composed mainly of a primary peak at a higher binding energy, attributed to fully condensed T^3^ network crosslinking points (C‐Si(‐O‐Si)_3_), and a secondary peak at a lower binding energy, which represents all incompletely condensed structures [38, 39]. These include T^2^ units bearing residual alkoxy or silanol groups, as well as C—O—Si linkages formed from the reaction between reactive hydroxyls and alkoxy groups during step 2 of the SAE synthesis. A comparison of the relative peak areas shows that the T^3^ structure is predominant in the PSAs, a finding that is consistent with the NMR results.

### 2.3 Thermostability

**FIGURE 3:**
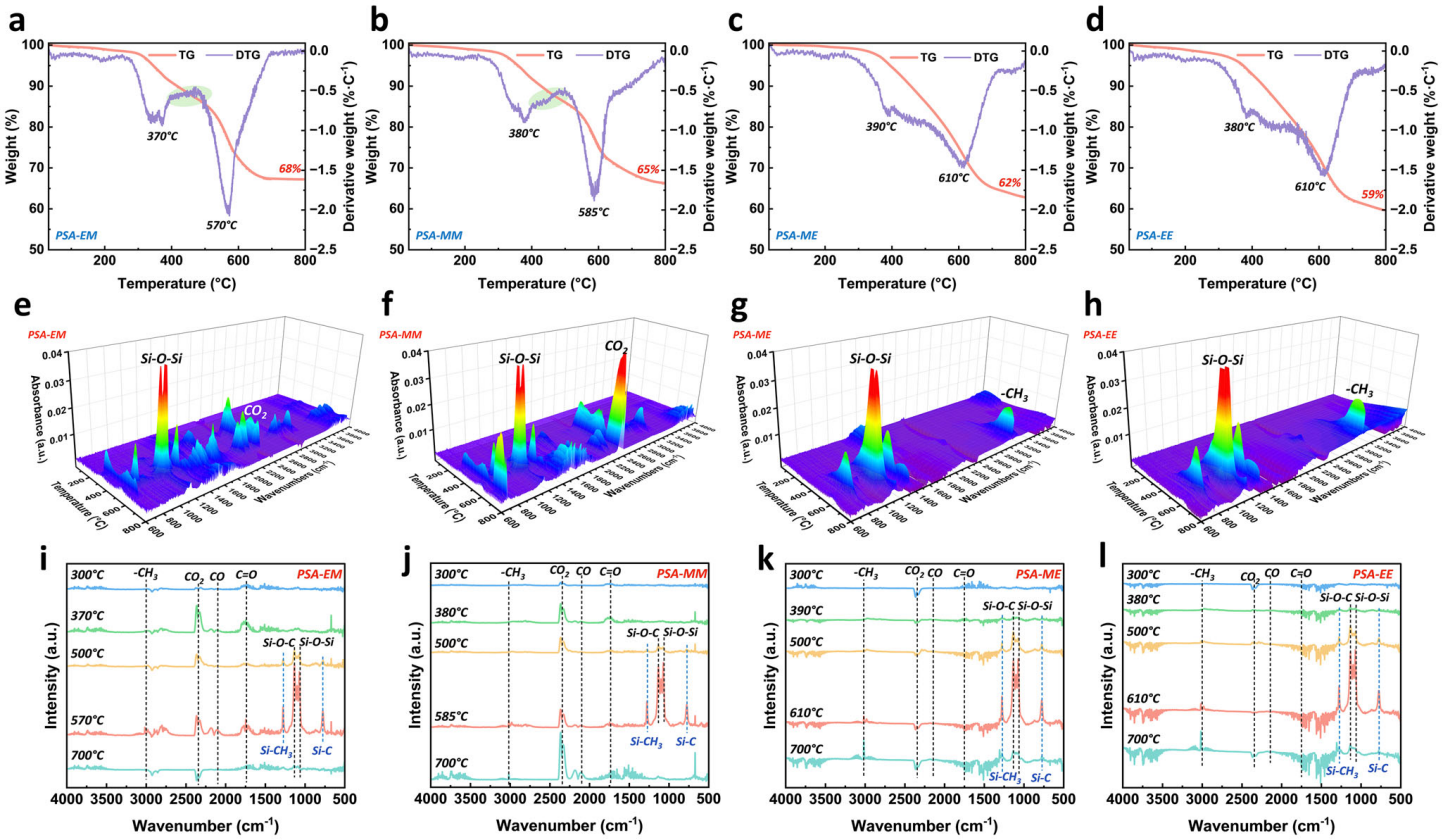
Thermostability. (a–d) TG and DTG curves of PSAs in N_2_: (a) PSA‐EM, (b) PSA‐MM, (c) PSA‐ME, and (d) PSA‐EE. (e–h) FT‐IR stacking plot of PSAs: (e) PSA‐EM, (f) PSA‐MM, (g) PSA‐ME, and (h) PSA‐EE. (i–l) FT‐IR spectra of gaseous products at different temperatures: (i) PSA‐EM, (j) PSA‐MM, (k) PSA‐ME, and (l) PSA‐EE.

The thermal stability of silicone aerogels is a critical performance indicator that directly influences their behavior in practical applications. Consequently, the thermal stability and decomposition pathways of the PSAs were investigated in detail. Under an air atmosphere, all variants exhibit a residual mass of approximately 78%, consistent with their similar inorganic (Si—O—Si) content (Figure S15). Notably, the DTG curves reveal that the oxidation rate for PSA‐EM around 300°C is markedly lower than that of the other samples. This is attributed to its higher degree of crosslinking, which imparts superior oxidation resistance and reduces its initial rate of thermal degradation [40]. In contrast, the pyrolysis processes of the PSAs under a nitrogen atmosphere show significant differences. A positive correlation is observed between the aerogel's degree of crosslinking and its char yield, which increases from 59% for PSA‐EE to a maximum of 68% for PSA‐EM (Figure 3a–d). This trend is a direct consequence of the orderly polymerization of the SAE‐EM precursor, which results in a superior network structure with fewer defects and thus a greater intrinsic resistance to thermal decomposition. These structural differences profoundly influenced the kinetic pathways of pyrolysis. The DTG curves for PSA‐EM and PSA‐MM display a distinct two‐stage decomposition profile. An initial peak in the decomposition rate occurs at ∼380°C, corresponding to the pyrolysis of organic side groups, followed by a noticeable decrease in the rate between 400–500°C. A second decomposition stage, attributed to the rearrangement and breakdown of the Si—O—Si framework, reaches its maximum rate at ∼580°C. In contrast, the decomposition rate for PSA‐ME and PSA‐EE increases continuously, peaking at a much higher temperature of ∼610°C. This profile indicates that the lower degree of crosslinking in PSA‐ME and PSA‐EE allows their Si‐O‐Si framework to undergo cleavage at much lower temperatures. This early onset of framework decomposition overlaps with the side‐group pyrolysis, resulting in a single, continuous increase in the decomposition rate that does not peak until late in the process. Furthermore, in the final decomposition stage (700−800°C), the pyrolysis of PSA‐EM is complete, with no further significant mass loss, whereas the other samples continue to exhibit substantial mass loss, providing additional evidence for the superior thermal stability of the PSA‐EM skeleton.

These proposed pyrolysis mechanisms are further elucidated by coupled TG‐FTIR, which provides online monitoring of the evolved gaseous products. As shown in Figure 3e–h, the evolved products for all samples primarily originate from two sources: small molecules from the cleavage of organic groups, and products from the cleavage and rearrangement of the Si‐O‐Si framework. However, the specific pyrolysis behavior varies significantly among the different PSAs. As shown in Figure 3i–l, below 300°C, decomposition primarily involves the condensation of residual silanol groups and some partial degradation of organic structures [12, 41], evolving small molecules such as H_2_, H_2_O, CO/CO_2_, and CH_4_. Significant distinctions appear at the first major decomposition peak (∼380°C), where PSA‐EM and PSA‐MM generate a large amount of CO_2_, whereas PSA‐ME produces only a trace amount, and the CO_2_ signal for PSA‐EE is weaker still. This is because methoxy groups tend to cleave and form CO_2_ via surface‐catalyzed reactions, whereas ethoxy groups are more inclined to form C_2_ fragments [42]. Crucially, at this same temperature, PSA‐ME and PSA‐EE already exhibit a distinct absorption band (∼1080 cm^−^^1^) corresponding to Si‐O‐Si framework cleavage. This finding provides direct evidence that their more defective networks undergo main‐chain decomposition at an earlier stage, which is consistent with the TG results. As the temperature rose into the 500–600°C range, all samples exhibit extensive cleavage and rearrangement of the Si‐O‐Si framework, which is the primary cause of mass loss in this stage. By 700°C, the pyrolysis of PSA‐EM is complete, with negligible subsequent mass loss. In contrast, the other samples continue to decompose vigorously: PSA‐MM still generates substantial amounts of CO_2_, while PSA‐ME and PSA‐EE continue to produce significant quantities of methane and species containing Si‐CH_3_ groups from the cleavage of the PMS backbone [41]. These results collectively provide further evidence for the outstanding high‐temperature thermal stability of PSA‐EM.

Coupled GC/MS was used as a complementary tool to analyze the higher molecular weight products (Mw ≥ 100) evolved between 500°C and 800°C, with the results and relative contents (RC) presented in Figure S16 and Tables S4–S7. The pyrolysis products are generally consistent across all samples, indicating structural similarities in their backbones, and can be classified into three groups based on their retention times (RT). The first group (0–10 min) consists of ester compounds, with methyl methacrylate as a major product, which originate from the combination of fragments from the SAE precursor and methyl radicals from Si‐CH_3_ group cleavage. The second group (10–20 min) is primarily octamethylsilsesquioxane (POSS), formed by the cleavage, rearrangement, and subsequent cyclization of the Si‐O‐Si framework [40]. The third group (20–25 min) is composed of long‐chain hydrocarbon products (C ≥ 17) that form from fragments of the DBTL catalyst. However, a key difference is noted: the relative content of POSS is significantly higher in the pyrolysis products of PSA‐ME and PSA‐EE compared to the other two samples. This finding confirms a more drastic decomposition of the Si‐O‐Si main chain in these samples, aligning with the preceding analysis. In summary, this body of evidence demonstrates that the PSAs prepared from different precursors follow distinct pyrolysis pathways, and the superior degree of crosslinking and structural integrity of PSA‐EM endows it with the highest overall thermal stability.

**FIGURE 4:**
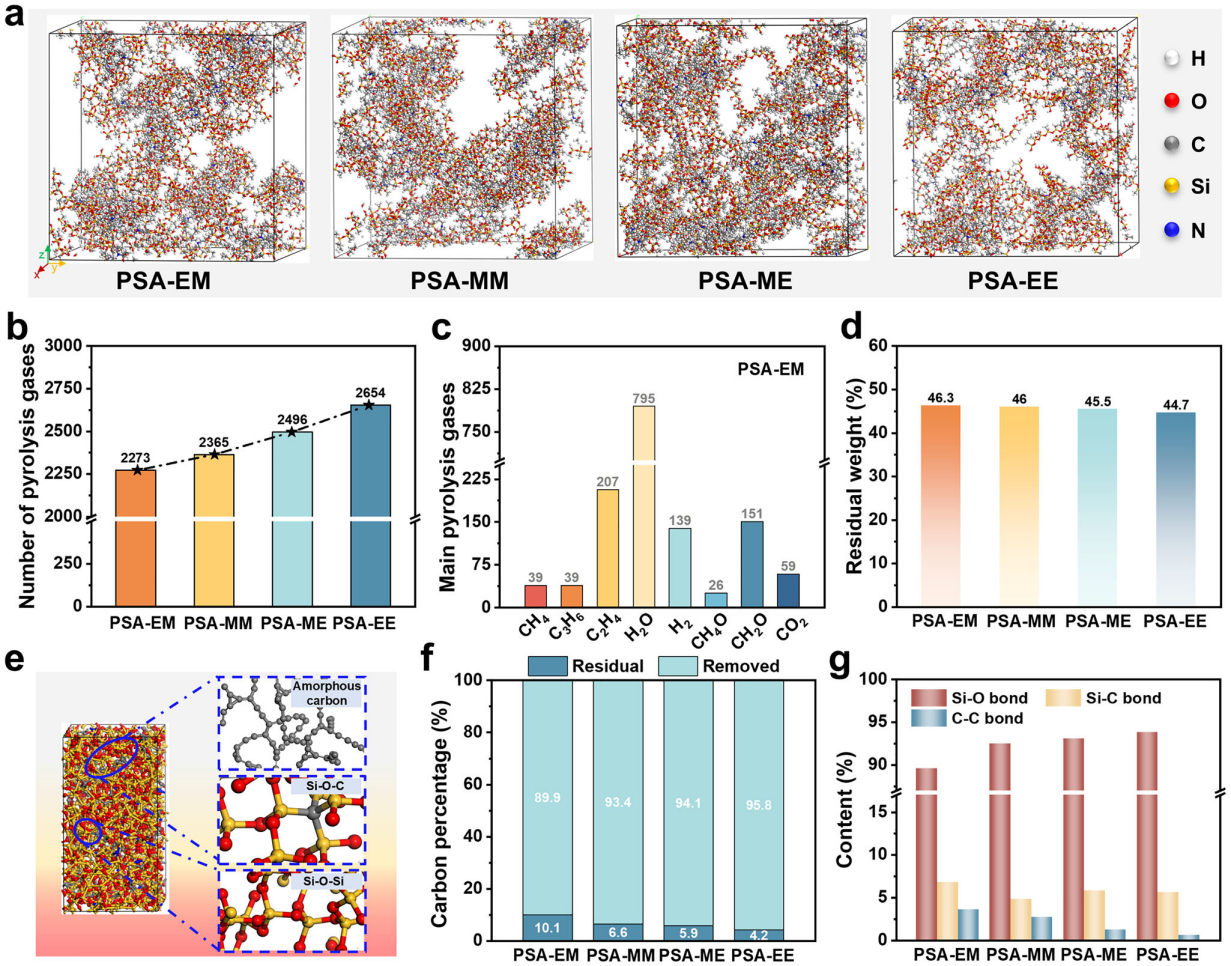
ReaxFF MD simulation. (a) Molecular model of different PSAs. (b) Number of pyrolysis gases. (c) Main types and numbers of pyrolysis gases of PSA‐EM. (d) Residual weight. (e) Structure of solid‐phase products. (f) Carbon content in the organic phase. (g) Contents of Si—O, Si—C, and C—C bonds in solid‐phase products.

To further probe the differences in thermal stability among various PSAs, we conducted ReaxFF MD simulations to reveal and track the characteristics of pyrolysis gases and solid‐phase products at the molecular level. The pyrolysis temperature in ReaxFF MD was set to 3400 K to accelerate reaction kinetics and compensate for the limited simulation timescale. It should be noted that, under such highly accelerated conditions, the simulations are not intended to quantitatively reproduce the absolute decomposition temperature, residue yield, or exact product distribution observed in the TGA experiments. Instead, they are used to provide qualitative insight into the relative thermal stability and plausible bond‐breaking pathways of the different PSA networks [40]. Based on the PSA structural units shown in Figure S17, molecular models of different PSAs were constructed (Figure 4a), with detailed information provided in Table S8. Simulations were performed following the pyrolysis process described in Figure S18 for the PSAs models. As shown in Figure 4b, the release amounts of pyrolysis gases for PSA‐EM, PSA‐MM, PSA‐ME, and PSA‐EE are 2273, 2365, 2496, and 2654, respectively, indicating an increasing trend. Figure 4c and Figure S19 detail the types and numbers of major pyrolysis gases, identifying CH_4_, C_3_H_6_, C_2_H_4_, H_2_O, H_2_, CH_4_O, CH_2_O, CO, and CO_2_. Notably, the simulated evolution of hydrocarbon gases derived from side‐group scission is qualitatively consistent with the specific absorption bands observed in the experimental TG‐FTIR spectra, supporting the plausibility of the bond dissociation pathways predicted by the model. Furthermore, PSA‐MM generates the most CO_2_, followed by PSA‐EM, PSA‐ME, and PSA‐EE, suggesting that a higher methoxy group content results in more CO_2_ production, which is consistent with the specific peak intensity variations in the TG‐FTIR results.

Regarding the solid residues, Figure 4d presents the residual weights of the solid‐phase products (detailed in Figure S20) for PSA‐EM (46.3%), PSA‐MM (46.0%), PSA‐ME (45.5%), and PSA‐EE (44.7%), indicating a thermal stability order of PSA‐EM > PSA‐MM > PSA‐ME > PSA‐EE. This simulated stability hierarchy is qualitatively consistent with the experimental TGA trend suggesting that the kinetic control strategy effectively enhances the thermal resistance of the bulk material. Statistical analysis of the solid‐phase product structures shows that they mainly consist of amorphous carbon, Si‐O‐C, and Si‐O‐Si structures, as illustrated in Figure 4e. To further elucidate the structural differences in PSA solid‐phase products, we studied the changes in organic carbon content (Figure 4f) and the composition of chemical bonds (Figure 4g). Figure 4f demonstrates that the organic carbon content removal rate increases and the residual rate decreases across the PSA‐EM, PSA‐MM, PSA‐ME, and PSA‐EE systems, with the PSA‐EM retaining a relatively high organic carbon content of 10.1%. In Figure 4g, the proportion of Si─O bonds in the pyrolysis solid‐phase products exceeds 89%, Si‐C bonds account for approximately 5%–7%, and C—C bonds for approximately 1%–4%. As the system changes from PSA‐EM, PSA‐MM, PSA‐ME to PSA‐EE, the proportion of Si—O bonds gradually increases, the proportion of Si—C bonds remains stable, and the proportion of C—C bonds decreases (consistent with the trend in Figure 4f). These results suggest that PSA‐EM, due to its higher degree of cross‐linking, exhibits superior thermal stability and retains a higher organic carbon content.

### 2.4 Mechanical and Thermal Insulation Properties

**FIGURE 5:**
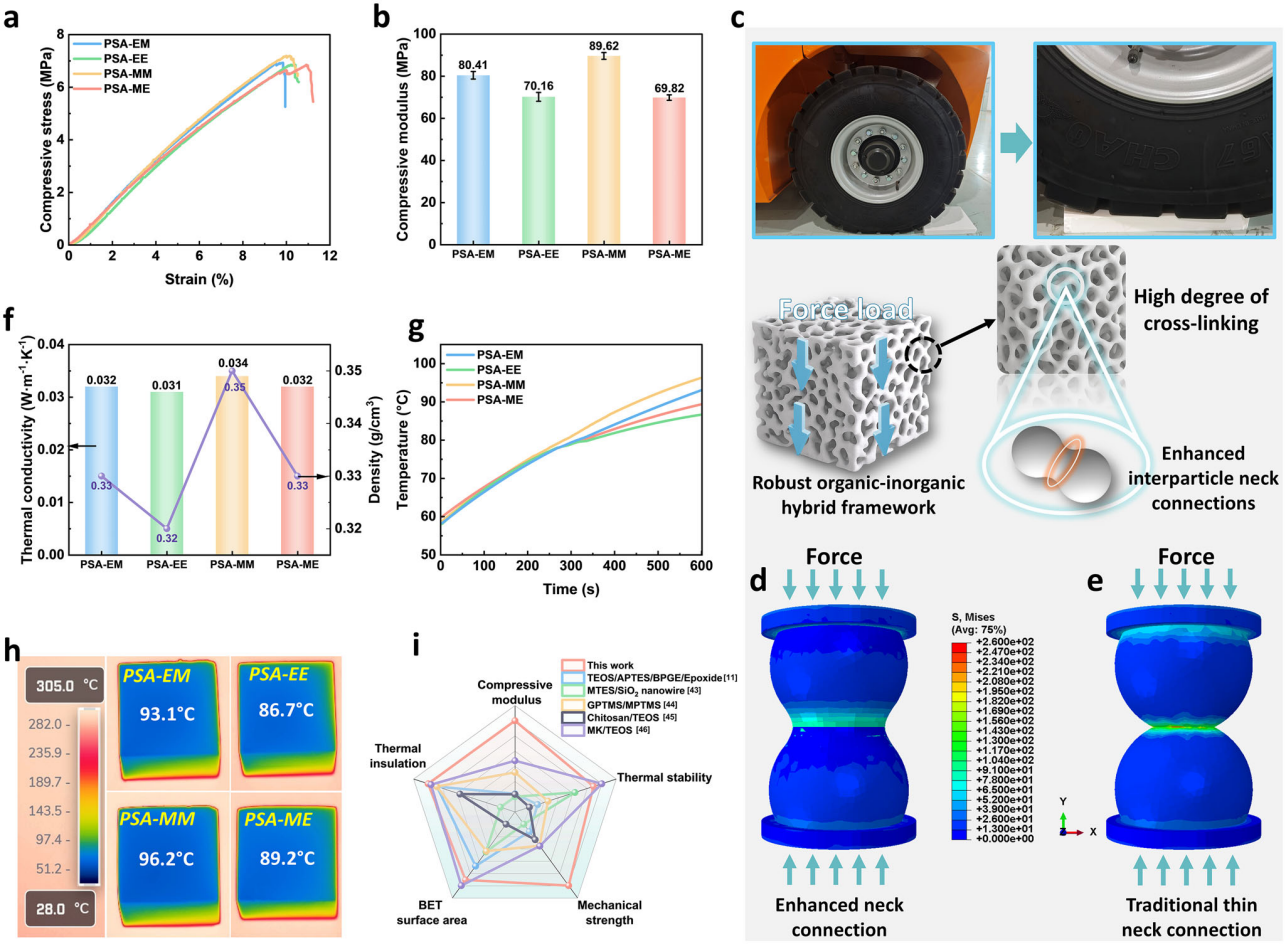
Mechanical and thermal insulation properties. (a) Compressive stress–strain curves of PSAs. (b) Compression modulus of PSAs. (c) Photograph of the PSA‐EM aerogel being run over by a forklift and a schematic diagram of its high‐strength mechanism. (d and e) Finite element simulations of thin and enhanced neck connection for the particles aggregated aerogels, illustrating stress distribution upon a force of compression. (f) Density and thermal conductivity of PSAs. (g) Time‐dependent temperature curves at a point on the back side of PSAs. (h) A pseudo color thermal image after heating 10 min. (i) Radar chart comparing the properties of PSA with other aerogels [10, 43, 44, 45, 46].

Superior mechanical strength is fundamental for the reliable processing and service of aerogel materials in practical applications. PSAs in this study exhibit remarkable mechanical performance owing to their unique organic‐inorganic hybrid network. As illustrated by the compressive stress‐strain curves in Figure 5a, all PSA samples achieve a compressive strength of approximately 7.1 MPa. Notably, PSA‐MM displays the highest compressive modulus, reaching 89.62 MPa, which is attributed to its higher shrinkage and density (Figure 5b). This combination of high strength and exceptional toughness is visually demonstrated in Figure 5c, where a PSA‐EM sample maintains structural integrity without catastrophic failure after being subjected to a 5‐ton forklift (Figure S21). This performance starkly contrasts with the inherent brittleness of conventional silica aerogels.

The unique performance of PSA‐EM originates from a cascading reinforcement mechanism across scales. At the nanoscale, hyperbranched SAE and linear PMS, rich in reactive sites, undergo co‐condensation, wherein PMS bridges hyperbranched SAE crosslinking nodes. This process transforms the fragile “point‐contact” particle necks typical of traditional aerogels into a robust, continuous “thick‐neck” architecture [18]. This thickened framework serves as the primary load‐bearing unit, preventing the collective collapse of nanoparticles. However, a purely rigid thick‐neck structure would still be prone to brittle failure; thus, the molecular‐scale flexible segments are critical for long‐term reliability. Under stress, these flexible segments absorb and dissipate energy through conformational changes, inhibiting stress concentration and crack propagation, thus imparting intrinsic toughness [47]. Specifically, the hyperbranched SAE nodes act as “tough junctions” that covalently bridge rigid Si—O—Si chain with flexible organic segments, ensuring that applied loads are not only distributed across the macro‐network (nanoscale) but also effectively dissipated within the molecular chains. To further illustrate the mechanical effect of the interparticle neck geometry, finite element analysis (FEA) was performed on simplified particle‐aggregate models with different connection modes. The simulations were carried out using Abaqus, in which the thin‐neck and thick‐neck geometries were compared under the same loading condition using a simplified linear elastic model. One end of the model was fixed and a pressure of 0.1 MPa was applied to the opposite end. Full computational details are provided in the Supporting Information. As shown in Figure 5d,e, the traditional point‐contact model exhibits severe stress concentration at the narrow neck region under compression, which serves as the potential initiation site for structural failure. In sharp contrast, the robust thick‐neck model representative of PSA demonstrates a uniform stress distribution with significantly reduced local stress levels. These results qualitatively suggest that the reinforced neck structure is more effective in dispersing the applied load and mitigating local stress concentration, thereby providing mechanistic support for the enhanced mechanical strength of PSAs.

Room‐temperature thermal conductivity (RTC) is a critical metric for evaluating insulation performance. As shown in Figure 5f, all PSA samples have a density of approximately 0.32 g·cm^−3^ and exhibit RTC values clustered around an comparatively low 0.032 W·m^−1^·K^−1^. PSA‐MM shows a slightly higher RTC, consistent with its marginally higher density. This excellent insulation performance results primarily from the aerogels’ abundant nanoporous structure. Complex solid–gas interfaces and tortuous heat transfer paths effectively suppress both gas‐phase and solid‐phase thermal conduction. Additionally, the intricate three‐dimensional skeletal network impedes heat transfer through strong phonon scattering [48]. To assess the PSAs’ potential in practical scenarios, their dynamic heat insulation performance was investigated. As shown in Figure 5g,h, when the samples were exposed to a constant hot‐side temperature of 300°C, the backside temperatures stabilized at 86–96°C after 10 min. This result demonstrates the outstanding heat‐blocking capability of PSAs. Notably, PSA‐MM exhibits the highest backside temperature (96.2°C), aligning with its slightly higher RTC. In contrast, PSA‐EE and PSA‐ME show slightly lower backside temperatures, likely due to their faster pyrolysis rates at this temperature. Endothermic decomposition reactions act as an ablative degradation mechanism, consuming some incoming thermal energy and temporarily reducing the backside temperature [49]. This behavior supports the earlier finding that these variants have relatively lower thermal stability.

To provide a clearer assessment of the comprehensive performance of PSAs, their key metrics are compared with those of other reported typical organic‐inorganic hybrid aerogels in Figure 5i [10, 43, 44, 45, 46]. Within a similar density range, the PSAs demonstrate a synergistic combination of superior mechanical strength and exceptionally low thermal conductivity. These attributes position PSAs as advanced materials with exceptional structural and functional integration, offering substantial advantages for high‐performance insulation and structural applications.

### 2.5 Evaluation of the Thermal Protection Performance of PSA‐Based Composites

**FIGURE 6:**
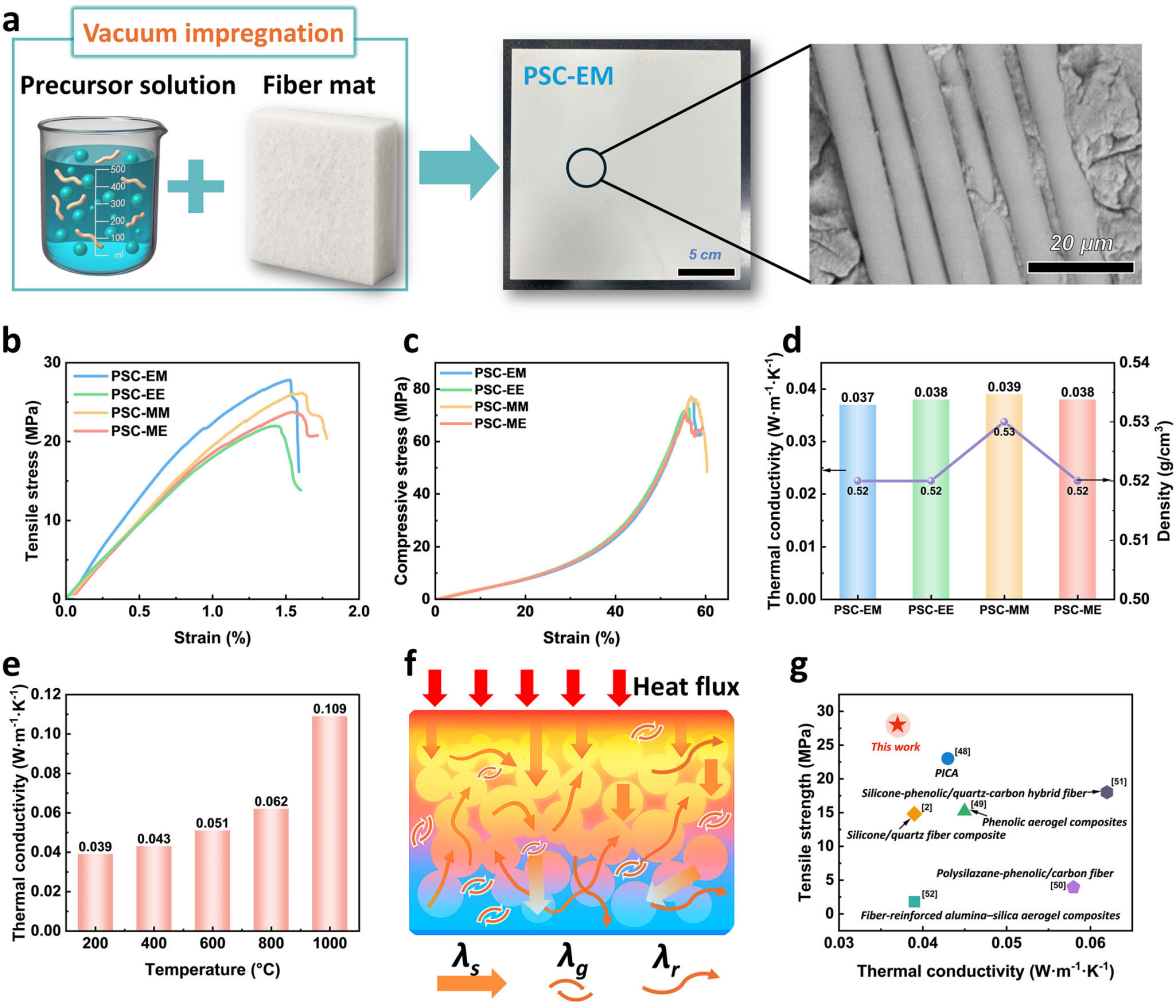
Mechanical and thermal insulation properties of PSCs. (a) Preparation process of PSCs. (b) Tensile stress–strain curves of PSCs. (c) Compression performance of PSCs. (d) Density and thermal conductivity of PSCs. (e) The thermal conductivities of PSC‐EM at different temperatures. (f) Schematic of heat transfer mechanism. (g) Tensile strength and thermal conductivity in this work compared with other aerogel composites [2, 50, 51, 52, 53, 54].

Given the excellent intrinsic mechanical, thermal insulation, and thermal stability properties of PSA, a fiber reinforcement strategy is key to further enhancing their comprehensive performance and expanding their practical applications. In this work, PSA‐based composites (PSCs) were fabricated by reinforcing low‐density quartz fiber mats with a PSA matrix through a scalable, cost‐effective sol‐impregnation and in‐situ gelation process (Figure 6a). Unlike the complex and costly manufacturing processes required for conventional rigid insulation tiles, this method offers significant advantages in scalability and cost‐efficiency. SEM image (Figure 6a) reveals a uniform PSA matrix filling the interstices of the quartz fiber network, with no visible pores or debonding at the fiber‐matrix interface. This seamless integration demonstrates strong interfacial compatibility and robust chemical bonding, providing a solid microstructural foundation for the composites’ superior mechanical properties.

Superior mechanical performance is a fundamental requirement for thermal protection materials in practical applications. The tensile and compressive properties of PSCs were systematically evaluated. Tensile stress–strain curves (Figure 6b) show that PSC‐EM achieves the highest tensile strength (28.1 MPa), while PSC‐EE exhibits the lowest (22.1 MPa). This variation stems from two key factors: the interfacial bonding strength between the matrix and fibers, and the intrinsic mechanical properties of the PSA matrix, both influenced by the reactivity of the precursor's alkoxy groups. Precursors with highly reactive methoxy groups (PSC‐EM and PSC‐MM) undergo more complete condensation with surface Si‐OH groups on quartz fibers, forming stronger chemical bonds that enhance stress transfer and yield higher tensile strengths compared to PSC‐ME and PSC‐EE. Additionally, PSC‐EM's superior performance is attributed to its optimized matrix network, which effectively disperses stress and inhibits microcrack propagation, maximizing tensile strength through synergy with the robust interface. In compression, PSCs display typical porous material behavior (Figure 6c), with an initial linear elastic phase followed by progressive pore compaction, enabling significant energy absorption without catastrophic failure. With similar densities across samples, their compressive strengths are also consistent, but PSC‐EM exhibits the highest modulus (Figure S22) due to its highly crosslinked network. Compared to brittle conventional silicon aerogel composites, PSCs combine exceptional strength and toughness, highlighting their potential for applications requiring both structural integrity and thermal protection [44, 55].

Benefitting from the excellent intrinsic insulation properties of the PSA matrix, PSCs likewise exhibit remarkable thermal insulation performance. All samples, with densities of approximately 0.52 g·cm^−3^, achieve a low room‐temperature thermal conductivity of ∼0.038 W·m^−1^·K^−1^ (Figure 6d). This relatively low thermal conductivity, despite the higher bulk density compared with ultralight monolithic aerogels, can be attributed to the preserved nanoporous structure of the aerogel phase within the quartz‐fiber framework, the low intrinsic thermal conductivity of the polysiloxane matrix, and the tortuous heat‐transfer pathways in the composite structure. Therefore, the density–thermal conductivity relationship in PSCs should be interpreted in the context of their hierarchical porous‐reinforced architecture rather than solely on the basis of bulk density.

The thermal conductivity of PSC‐EM was further measured across a temperature range, increases from 0.039 W·m^−1^·K^−1^ at 200°C to 0.109 W·m^−1^·K^−1^ at 1000°C (Figure 6f). This temperature‐dependent increase reflects a shift in dominant heat transfer mechanisms. At low temperatures, the small pore sizes of PSCs suppress gas‐phase conduction (λ_g_) and convection (λ_con_), with solid‐phase conduction (λ_s_, proportional to ρ^1–1.5^) dominating. At higher temperatures, radiative heat transfer (λ_r_, proportional to T^3^) becomes predominant, driving the rise in thermal conductivity [56, 57]. Nevertheless, PSCs maintain low thermal conductivity across a wide temperature range (room temperature to 1000°C), underscoring their efficacy as versatile thermal insulation materials. In summary, compared with reported aerogel composites in a similar application space [2, 50, 51, 52, 53, 54], PSCs offer a competitive balance between thermal insulation and mechanical robustness.

**FIGURE 7:**
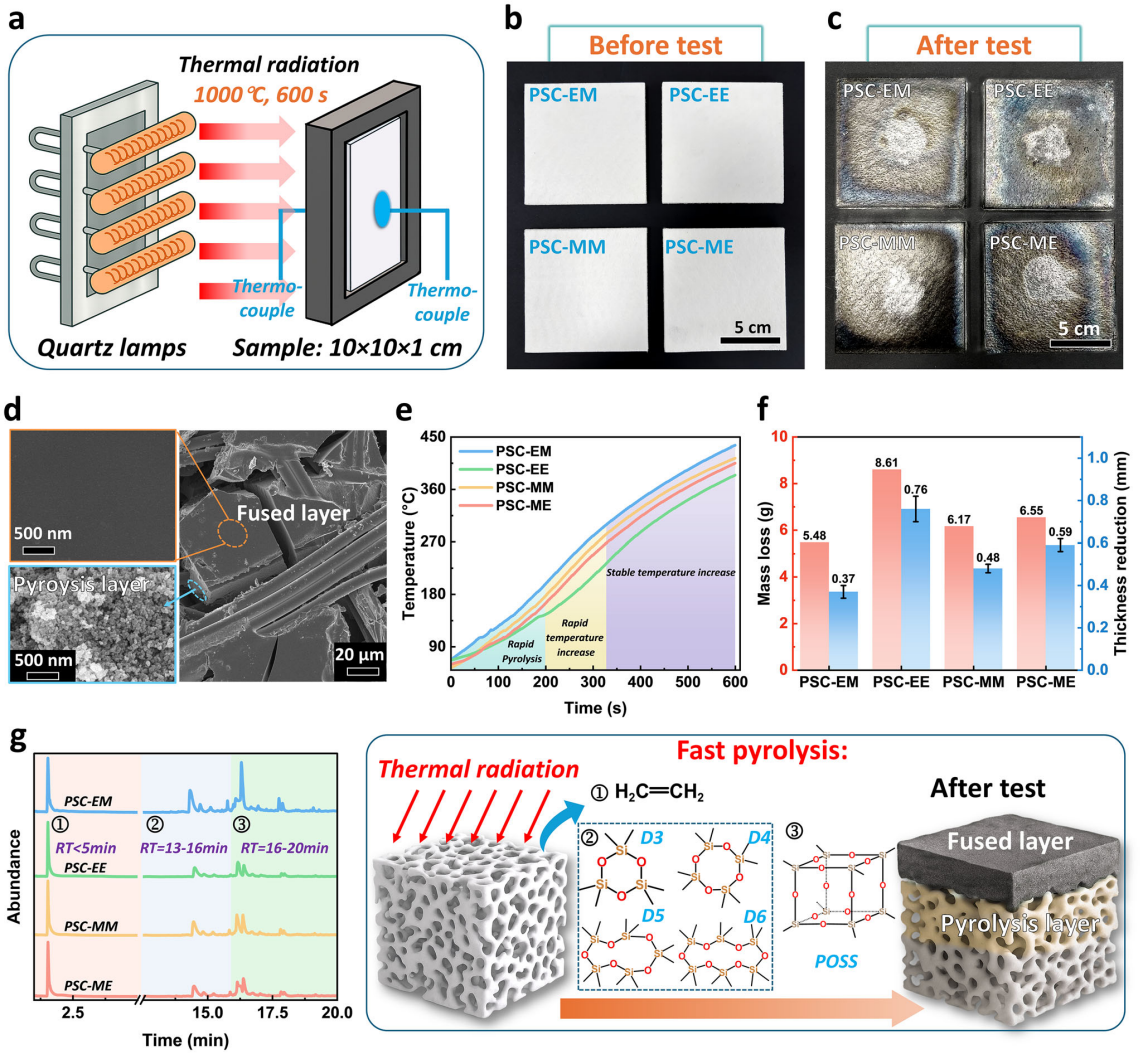
Evaluation of static thermal insulation properties under quartz lamp irradiation. (a) Schematic diagram of a static quartz lamp heating equipment. (b,c) Photographs of the heated surface of PSCs before and after testing. (d) SEM images of PSC‐EM after test. (e) Temperature variation curves of the back side of PSCs during the testing. (f) Mass loss and linear recession of the PSCs after quartz lamp testing. (g) The py‐GC/MS chromatogram of PSCs and schematic illustration of structural evolution during the test.

Effective thermal insulation at high temperatures is critical for thermal protection materials in practical applications. To assess the thermal insulation and structural stability of PSCs, a static heat flux test was conducted using a quartz lamp at 1000°C for 600 s in a nitrogen atmosphere (Figure 7a). Post‐test photographs (Figure 7b,c) reveal that the heated surfaces of all samples darkened, which is attributed to the matrix's transformation into a ceramic phase comprising free carbon, amorphous silica, and SiOC [38]. SEM observation of the post‐test microstructure (Figure 7d; Figures S23–S25) reveals a robust interfacial bond between the quartz fibers and the PSA matrix, with no evidence of debonding. However, significant microcracks are observed in the matrix, resulting from pyrolysis‐induced volatile release and thermal stress from the large temperature gradient. Notably, a dense, in‐situ‐formed fused layer on the outermost surface effectively impedes further heat transfer into the composite's interior, preserving the inner matrix's nanoporous structure and maintaining its structural integrity.

Dynamic changes in the backside temperature during the test are illustrated in Figure 7e. Within the first 200 s, temperature fluctuations occur due to rapid pyrolysis. From 200 s to 330 s, the backside temperature increases rapidly as a stable temperature gradient form. Subsequently, the heating rate slows as the temperature difference between the front and back surfaces diminishes. However, a notable variation is observed in the final backside temperatures: PSC‐EM reaches the highest (436°C), while PSC‐EE records the lowest (383°C). This difference correlates strongly with mass and thickness loss post‐test (Figure 7f). PSC‐EM, with its superior matrix thermal stability, exhibits the least mass loss and thickness recession, resulting in thermal insulation dominated by conduction and a higher backside temperature. In contrast, PSC‐EE's greater pyrolysis, acting as an endothermic ablative mechanism, dissipates significant heat, lowering its backside temperature.

To further elucidate the chemical mechanisms during rapid pyrolysis, Pyrolysis‐Gas Chromatography/Mass Spectrometry (Py‐GC/MS) analysis was performed at 1000°C (Figure 7g; Table S9). Unlike slow heating, fast pyrolysis produces ethylene as the primary small‐molecule product (RT < 5 min), indicating deep fragmentation of the organic structure at high temperatures. PSC‐EE yields the highest ethylene content (32.93%), while PSC‐EM produces the lowest (16.20%), confirming its superior organic structure stability. The Si‐O‐Si backbone also undergoes significant cleavage and rearrangement, with cyclic siloxanes dominating in range of 13–16 min, generated by the cyclization of the polymer backbone following chain scission. Given that cyclic trimers and tetramers are inherently thermodynamically stable at the degradation temperatures used, they constitute the major products of the reactions [40]. Products in the RT = 16–20 min, primarily octamethylsilsesquioxane (POSS), reflect the characteristic rearrangement of T‐unit networks at high temperatures. These findings underscore that PSC‐EM's highly crosslinked network confers exceptional structural stability in extreme thermal environments. Although its lower degree of pyrolysis leads to a slightly higher backside temperature, this is precisely a direct manifestation of its superior performance as a structurally and functionally integrated thermal protection material.

To comprehensively assess the performance of PSCs under extreme thermo‐mechanical environments, an oxy‐propane ablation test was conducted at 1000°C for 300 s, subjecting the samples to intense chemical pyrolysis and mechanical erosion (Figure S24). As shown in Figure S26, all PSC samples exhibit outstanding resistance to structural deformation under the severe thermo‐mechanical shock, and their backside temperatures remain at approximately 130°C after 300 s of continuous ablation, confirming their excellent thermal insulation capabilities (Figure S27c). However, variations in the intrinsic properties of the matrices lead to distinct ablation behaviors: PSC‐EM, leveraging its superior thermal stability and mechanical strength, demonstrates the highest ablation resistance, with a linear recession and mass loss of only 1.05 mm and 0.30 g, respectively. The microstructural evolution after ablation reveals the origin of this high performance: on the ablated surface, an in‐situ formed and continuous oxide layer is observed, which effectively retards heat diffusion into the interior. Meanwhile, in the pyrolysis layer beneath, the PSA matrix remains intimately bonded to fiber bundles without interfacial debonding, and its nanoporous structure is well‐preserved. This exceptional ablation resistance originates from the synergistic interaction between the high‐strength quartz fibers and the thermally stable silicone aerogel matrix, which endows the composite with high structural integrity and service reliability, positioning it as a highly attractive solution for ultra‐high‐temperature insulation in advanced industrial and aerospace applications.

## 3 Conclusion

In conclusion, we introduce a kinetically regulated synthetic strategy that enables the precise construction of a mechanically robust and thermally stable silicone aerogel (PSA) possessing hyperconnected network architecture. This strategy relies on the co‐condensation of rationally designed hyperbranched siloxane–amino/epoxy (SAE) nodes with linear polymethylhydrosiloxane (PMS). The synthesis of the hyperbranched SAE structure is governed by a temperature‐modulated amine–epoxy polycondensation, which deliberately exploits the kinetic disparity between methoxy‐ and ethoxy‐substituted silane precursors. This kinetic manipulation is proposed to effectively bias the reaction trajectory toward intermolecular crosslinking while suppressing intramolecular cyclization, thereby enabling homogeneous incorporation of flexible carbon‐chain segments alongside plentiful Si–OMe/Et reactive sites. Subsequent co‐condensation of the silica‐rich PMS with the hyperbranched SAE nodes produces a densely crosslinked PSA network, in which the uniformly distributed carbon‐chain segments are embedded within the silica backbone. This molecular architecture confers markedly enhanced mechanical strength and thermal stability, achieved with only minimal incorporation of organic components. Accordingly, the optimized PSA exhibits a compressive strength of 7.1 MPa, a compressive modulus of 80.41 MPa, a low thermal conductivity of 0.032 W·m^−1^·K^−1^ at room temperature, and a high char yield of 68% at 800°C in nitrogen. Moreover, the low‐density quartz fiber reinforced aerogel composite (PSC) achieves a tensile strength of 28.1 MPa and demonstrates outstanding high‐temperature insulation and ablation resistance up to 1000°C. This work presents a robust strategy for the design of advanced hybrid aerogels through precursor kinetics control, offering both a theoretical foundation and a practical synthetic blueprint for the development of next‐generation thermal insulation materials.

## 4 Experimental Section

### 4.1 Materials

Aminosilane monomers, including 3‐aminopropyltrimethoxysilane (APTMS) and 3‐aminopropyltriethoxysilane (APTES), and epoxysilane monomers, including 3‐glycidoxypropyltrimethoxysilane (EPTMS) and 3‐glycidoxypropyltriethoxysilane (EPTES), were purchased from Shanghai Macklin Biochemical Technology Co., Ltd. Anhydrous ethanol, distilled water, tetramethylammonium hydroxide (TMAH, 25 wt.% in methanol), and dibutyltin dilaurate (DBTL) were supplied by Shanghai Aladdin Biochemical Technology Co., Ltd. Polymethylhydrosiloxane (PMS, molecular weight ≈ 3000 g/mol, Si content: 29.5 wt.%) was purchased from Lota Silicone Oil Co., Ltd. Low‐density quartz fiber mat (density 0.20 g·cm^−3^) was obtained from Hubei Feilihua Quartz Glass Co., Ltd. All materials were used as received without further purification.

### 4.2 Preparation of SAE

As shown in Figure S1, the hyperbranched siloxane‐amino/epoxy resin (SAE) was synthesized via a temperature‐controlled sequential amine/epoxy addition‐polycondensation process. The initial step involved the addition reaction between amine and epoxy groups. To begin, an amino silane monomer and an epoxy silane monomer were mixed at a 1:2 molar ratio. Dibutyltin dilaurate (DBTL) was introduced as a catalyst at a concentration of 0.5% of the total reactant mass. The mixture was then held at 50°C for 2 h under a nitrogen atmosphere to ensure the complete ring‐opening of the epoxy groups, yielding a reaction intermediate. Subsequently, the temperature was elevated to 140°C and the reaction was continued for an additional 4 h to obtain the final hyperbranched SAE. The entire procedure was conducted under a protective nitrogen atmosphere to exclude air and water vapor. The detailed formulations and corresponding mass changes for the synthesis process are summarized in Table S1.

The synthesized SAEs, derived from various combinations of amino silanes and epoxy silanes, were designated using the nomenclature SAE‐XY. In this system, ‘X’ denotes the type of alkoxy group on the amino silane precursor, and ‘Y’ represents the alkoxy group on the epoxy silane precursor. Specifically, the letter ‘M’ corresponds to a methoxy group, while ‘E’ corresponds to an ethoxy group. Based on this combinatorial approach, four distinct SAE variants were synthesized and are referred to as SAE‐EM, SAE‐EE, SAE‐MM, and SAE‐ME.

### 4.3 Preparation of PSA

The hyperbranched SAE derived silicone aerogels (PSA) were prepared via a sol‐gel method followed by drying at ambient pressure. In a typical synthesis, 3.5 g of SAE and 10.5 g of PMS were dissolved in 26 g of ethanol. The mixture was stirred for 10 min at ambient temperature to ensure homogeneity. Subsequently, 0.73−0.87 g of deionized water was added. The precise amount of water was calculated based on the stoichiometric quantity required for the complete hydrolysis of the alkoxy groups present in each specific SAE variant; the exact water quantities used for each formulation are detailed in Table S2.

The resulting solution was then stirred in a thermostatic water bath at 40°C for 2 h to facilitate pre‐hydrolysis. After the solution had cooled to room temperature, 0.22 g of TMAH was slowly introduced, and the mixture was stirred for an additional 10 min, yielding a clear precursor solution for the sol‐gel process. This solution was then cast into a sealed mold and subjected to a multi‐stage curing process: 12 h at 60°C, followed by 12 h at 100°C, and finally 12 h at 150°C to obtain wet gel. The final PSA product was obtained after a two‐step ambient pressure drying schedule: 12 h at 60°C and then 12 h at 100°C. The obtained aerogels were designated as PSA‐XY, where XY corresponds to the type of SAE precursor used (i.e., PSA‐EM, PSA‐EE, PSA‐MM, and PSA‐ME).

### 4.4 Preparation of PSC

The PSCs were fabricated through a process combining vacuum impregnation and ambient pressure drying. First, a quartz fiber preform was placed into a mold. The transparent sol, prepared using the method described for the PSAs, was then injected into the mold. To ensure stable and uniform impregnation, the process was assisted by a vacuum, with the differential pressure maintained between 0 and 1 bar. As the mold approached full saturation, an additional pressure of 1–3 bar was applied to facilitate complete impregnation. This impregnation process was repeated until the fiber‐reinforced structure was thoroughly saturated with the sol, at which point the mold was hermetically sealed. Finally, the sealed molds were subjected to the same multi‐stage curing and drying protocols detailed for the PSAs to yield the final PSCs. The composites were named using the same convention as the PSAs.

### 4.5 Material Characterizations

The bulk density of aerogels was determined by measuring the mass and edge length of the cubic samples. The morphologies of the samples were observed by field emission scanning electron microscopy (FESEM, Helios G4 UC, USA) and field emission transmission electron microscopy (FETEM, JEOL 2100F, Japan). The porous structure was characterized with an N_2_ adsorption/desorption analyzer (3Flex, USA), the specific surface area (SSA) was obtained from the adsorption branch using the Brunauer–Emmett–Teller (BET) model, and pore size distribution was derived from the desorption branch using the Barrett–Joyner–Halenda (BJH) model.

The chemical structure of the samples was characterized by Fourier transform infrared spectroscopy (FTIR, Spectrum 100, USA) and nuclear magnetic resonance (NMR) spectroscopy. Specifically, liquid ^1^H NMR spectra for the liquid samples were recorded on a Bruker 400M spectrometer (Germany). Solid‐state ^29^Si magic angle spinning (MAS) NMR measurements for the aerogel samples were performed on a JEOL JNM‐ECZ600R spectrometer (Japan) using a single‐pulse sequence without paramagnetic relaxation reagents, acquiring 1024 scans with a relaxation delay of 3 s. The phase compositions and surface elemental compositions of the samples were analyzed by X‐ray diffraction (XRD, D/max 2550, Japan) and X‐ray photoelectron spectrometry (XPS, Thermo Kalpha, USA). Contact angles of water were measured using a contact angle tester (JC2000, China). The viscosity of the samples was measured using a rheometer (Thermo Scientific HAAKE MARS 60, USA) at a constant temperature of 25°C. The relative molar content of methanol and ethanol in the distillates from the SAE synthesis process was determined by gas chromatography (GC, Thermo Scientific TRACE 1300, USA).

The non‐isothermal polymerization kinetics of the four SAE precursors were investigated using Differential Scanning Calorimetry (DSC) on a Netzsch 200F3 analyzer. Samples (approx. 5–10 mg) were heated from 30°C to 200°C at a constant heating rate of 10 °C/min under a nitrogen atmosphere. The apparent activation energy (*E_a_*) was determined using the Horowitz‐Metzger (HM) method; the specific calculation method is detailed in Part B of supporting information.

The mechanical test was conducted using an Instron 3367 universal test machine. The thermal conductivity at room temperature was determined using a heat flow meter (HFM 446 Lambda, Netzsch, Germany). Prior to testing, the samples were cut into regular shapes (200 × 200 × 10 mm) and the testing surfaces were polished to improve flatness and parallelism. During measurement, a compressive load of 10 kPa was applied to improve the thermal contact between the sample and the instrument plates. Each sample was measured three times on both sides, and the averaged value was reported.

To assess the thermal insulation performance in practical applications, samples with dimensions of 20 mm × 20 mm × 15 mm were heated to a temperature of 300 °C using a photothermal heating system. and the temperature of the back side of the sample was recorded by a thermal infrared imager (FLIR T540, USA). The thermal conductivity at high temperatures was measured using the Hot Disk TPS 2500S (Sweden), with the testing environment being under nitrogen gas.

Evolved gas analysis throughout the decomposition process was conducted using a system coupling a thermogravimetric analyzer (TGA; PerkinElmer TG 8000) with a Fourier‐transform infrared spectrometer (FT‐IR; PerkinElmer Spectrum Two). During the analysis, samples were heated from 30°C to 800°C at a constant rate of 10 °C/min under a nitrogen atmosphere. The composition of the pyrolysis gases generated within the 500°C to 800°C temperature range was subsequently identified using a gas chromatography‐mass spectrometry (GC‐MS) system (PerkinElmer Clarus SQ8).

The ablation test was conducted using an oxygen‐propane torch test at 1000°C, with an ablation duration of 300 s. More details of the ablation test can be found in Part A of supplementary information.

The thermal insulation performance was investigated using a static quartz lamp apparatus under controlled high‐temperature conditions. The sample with typical dimensions of 100 mm × 100 mm × 10 mm was heated at 1000°C for 600 s under a nitrogen atmosphere. To elucidate the composition of the volatile pyrolysis products, Pyrolysis‐Gas Chromatography/Mass Spectrometry (Py‐GC/MS) analysis was conducted. Samples were pyrolyzed at a designated temperature of 1000 °C utilizing an EGA/PY‐3030D micro‐furnace pyrolyzer (Frontier Laboratories Ltd, Japan). The resultant volatile species were subsequently separated and identified employing a GC/MS‐QP2010 Ultra gas chromatograph‐mass spectrometer (Shimadzu Corporation, Japan).

### 4.6 Computational Details

All pyrolysis and carbonization simulations were conducted using the Large‐scale Atomic/Molecular Massively Parallel Simulator (LAMMPS) with the Velocity‐Verlet time integration algorithm. The Qeq charge equilibration method was employed to calculate and update atomic charges. Notably, a Nosé‐Hoover thermostat and barostat with damping coefficients of 100 fs and 1000 fs, respectively, were used to control the temperature and pressure of the simulation system. The simulations employed the CHONSSiNaP‐tribology force field parameter set with a time step of 0.1 fs to model the continuous formation and breaking of chemical bonds in the PSAs systems. Detailed discussion of the accelerated ReaxFF MD conditions and their qualitative relevance to the experimental pyrolysis trends is provided in Supporting Information Part C.

More details of the pyrolysis and carbonization processes are described in Figures S17–S20.

## Conflicts of Interest

The authors declare no conflict of interest.

## Supporting information




**Supporting File**: advs75069‐sup‐0001‐SuppMat.docx.

## Data Availability

The data that support the findings of this study are available from the corresponding author upon reasonable request.
