# Supplementary material for: Robust and Thermally Stable Silicone Aerogels with Hyperconnected Network via Kinetically Optimized Hyperbranched Silane Precursors
Source: Adv Sci (Weinh). 2026 Mar 31;13(34):e75069. doi: 10.1002/advs.75069 (PMC13285139; doi:10.1002/advs.75069)
Supplement: Supplementary file 1 — Supporting File: advs75069‐sup‐0001‐SuppMat.docx. [file ADVS-13-e75069-s001.docx]

**Supporting Information**

**Robust and** **Thermally Stable Silicone Aerogels with hyperconnected network via Kinetically Optimized Hyperbranched Silane Precursors**

*Aoqing Yan, ^a, b, 1^ Guixiang Li, ^b, 1^ Yaolan Li, ^a^ Yi Luo, ^b^ Bin Liu, ^a^ Zhe Su,* *^b^* *Hao Tian, ^a^ Wei Shi, ^a^ Bo Niu ^a, *^ and Donghui Long ^a, b, *^*

^a^ Key Laboratory of Specially Functional Polymeric Materials and Related Technology (Ministry of Education), School of Chemical Engineering, East China University of Science and Technology, Shanghai 200237, China.

^b^ Suzhou Laboratory, No.388, Ruoshui Street, SIP, Jiangsu 215123, China

^1^ These authors contributed equally to this work.

* Corresponding author: [longdh@mail.ecust.edu.cn](mailto:longdh@mail.ecust.edu.cn) (D.H. Long) and [niubo@ecust.edu.cn](mailto:niubo@ecust.edu.cn) (B. Niu)

**Part A: Detailed information of ablation test**

Here, the size of ablation specimen was Φ30 × 10 mm. The tests were performed with 5 parallel specimens to ensure statistical significance. A K-type thermocouple was utilized to record thermal responses on the backside surfaces. The linear ablation rate (R_l_) and mass ablation rate (R_m_) were calculated by employing the following formulas: ^[1]^

Where $l_{0}$ and $m_{0}$ represent the thickness and mass before ablation, $l_{t}$ and $m_{t}$ represents the thickness and mass after ablation, and $t$ is the ablation time.

**Part B: Detailed information of apparent activation energy calculation**

The non-isothermal curing kinetics were analyzed to determine the apparent activation energy ($E_{a}$) for the polymerization of the four SAE precursors (SAE-EM, SAE-EE, SAE-MM, and SAE-ME).

**1. Rationale for Method Selection**

The Horowitz-Metzger (HM) method, a single-heating-rate model, was employed for this analysis. While multi-heating-rate methods (e.g., Kissinger) are suitable for simple processes like crystallization, they are ill-suited for complex polymerization reactions. For such reactions, the polymerization mechanism itself (e.g., the competition between intermolecular crosslinking and intramolecular cyclization) can be dependent on the heating rate ($\beta$). This often leads to significant distortion of the DSC curves at different heating rates, invalidating the core assumptions of multi-rate models. The HM method bypasses this issue by analyzing a single, consistent DSC curve obtained at a constant heating rate ($\beta$ = 10 K/min), ensuring a self-consistent kinetic pathway.

**2. Calculation Method**

The Horowitz-Metzger equation^[2]^ is derived from the general rate equation. For a reaction at a constant heating rate, the equation can be approximated as:

$$\begin{aligned} \ln\left( G\left( \alpha\right) \right)=ln\left[ \frac{ART_{s}^{2}}{\beta E_{a}} \right]-\frac{E_{a}}{RT_{s}}+\frac{E_{a}\theta}{RT_{s}^{2}}\#\left( 1 \right) \end{aligned}$$

Where:

$G\left( \alpha\right)$ is the integral form of the conversion function.

$A$ is the pre-exponential factor.

$R$ is the ideal gas constant (8.314 J·mol⁻¹·K⁻¹).

$\beta$ is the constant heating rate (10 K/min).

$T_{s}$ is the characteristic temperature (in Kelvin) at which the fractional conversion $\alpha$ is 0.3679 (i.e., $1-\frac{1}{e}$).

$\theta$ is the temperature difference, $T-T_{s}$.

This equation takes the form of a linear relationship, $y=mx+c$:

$$\begin{aligned} \ln\left( G\left( \alpha\right) \right)\approx const+\left( \frac{E_{a}}{RT_{s}^{2}} \right)\theta\#\left( 2 \right) \end{aligned}$$

The apparent activation energy ($E_{a}$) can be reliably calculated from the slope ($m=E_{a}/RT_{s}^{2}$) of the linear plot of $\ln\left( G\left( \alpha\right) \right)$ versus $\theta$.

**Part C: Detailed Rationale for Simulation Temperature**

There is a significant difference between the timescales of laboratory experiments (minutes/hours) and molecular dynamics (MD) simulations (nanoseconds). To overcome the energy barriers for bond cleavage within the accessible computational window, elevated temperatures are standardly employed in ReaxFF MD. Such an approach has been widely used in previous studies to provide qualitative insight into thermally induced structural evolution.^[3–6]^

To provide an empirical reference for the physical relevance of the simulation temperature used in this work, we employed an empirical relationship proposed by Xia et al., which relates the simulation temperature (T_s_, K) to the corresponding experimental temperature (T_r_, K) based on solid residue yields:

$T_{r}=\frac{T_{s}+3087.14}{7.94}$ (1)

For T_s_ = 3400 K, the calculated T_r_ is approximately 817 K. This value falls within the experimental pyrolysis window observed in our TG-FTIR-GC/MS analyses and therefore provides a rough empirical indication that the accelerated simulation temperature is physically relevant at the trend level. However, this correspondence should not be interpreted as a direct quantitative mapping between the ReaxFF MD conditions and the experimental pyrolysis process.

**Part D: Detailed Information for Finite element analysis (FEA)**

FEA simulations were performed using the commercial software Abaqus. The model was constructed using the Revolution module, with the key geometric parameters shown in the corresponding figure. The short-neck and long-neck connection lengths were set to 4 and 7, respectively. To isolate the effect of geometry, it was assumed that the simulation results were only dependent on the model geometry. The loading surface and particles were assumed to be made of the same homogeneous isotropic linear elastic material, with a density of 1.2 g cm⁻³, a Young’s modulus of 2 GPa, and a Poisson’s ratio of 0.35. During the simulation, one end of the model was fully constrained, while a pressure of 0.1 MPa was applied to the opposite end. A global mesh size of 0.5 was adopted for mesh discretization. The simulations were intended to provide a qualitative comparison of stress distributions in models with different neck geometries.

**Part E: Supplementary Figures and Tables**


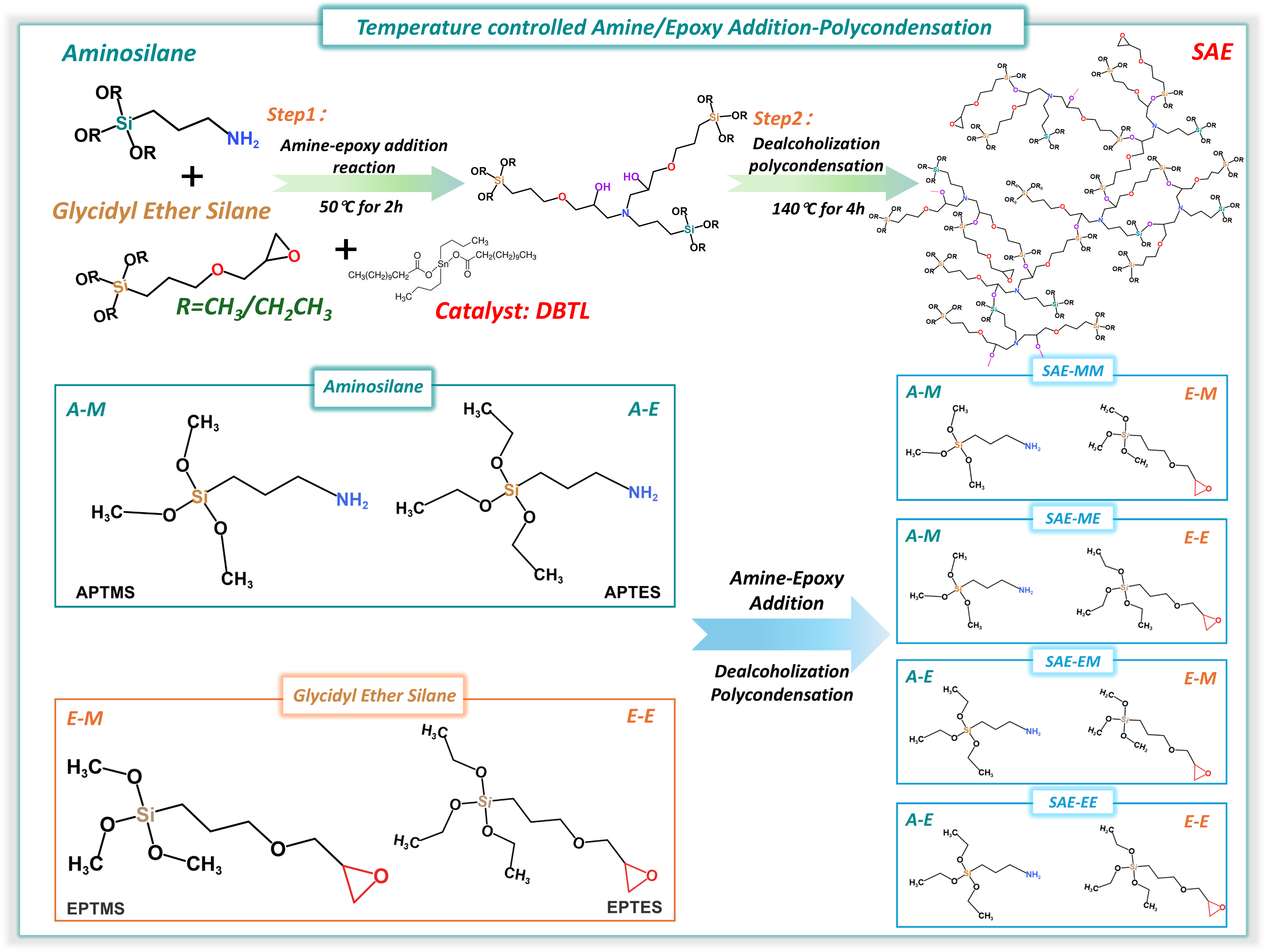


**Figure S1. Schematic of the synthesis route for SAE**.

**
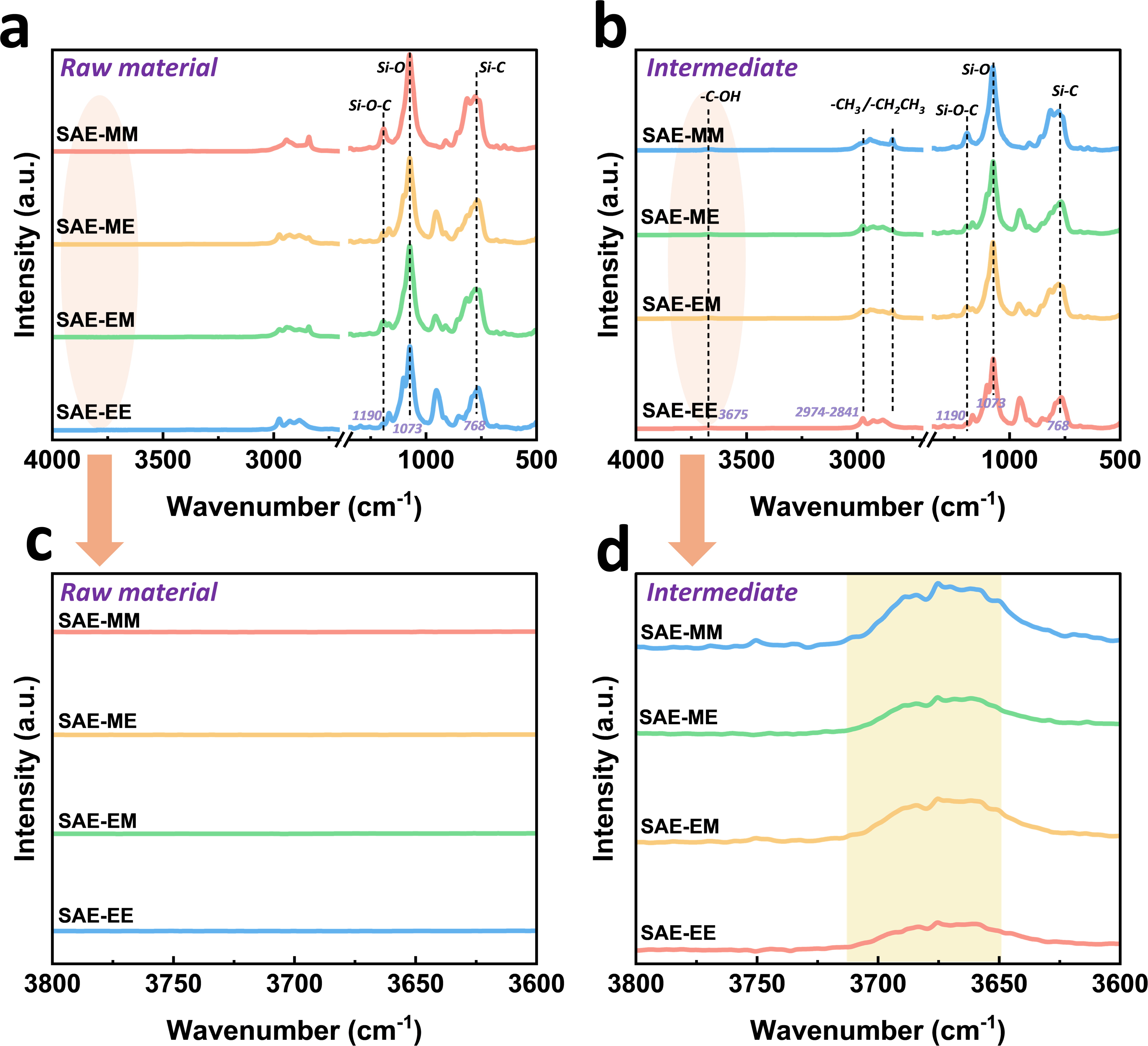
**

**Figure S2. FT-IR spectra of the raw material and intermediate.**


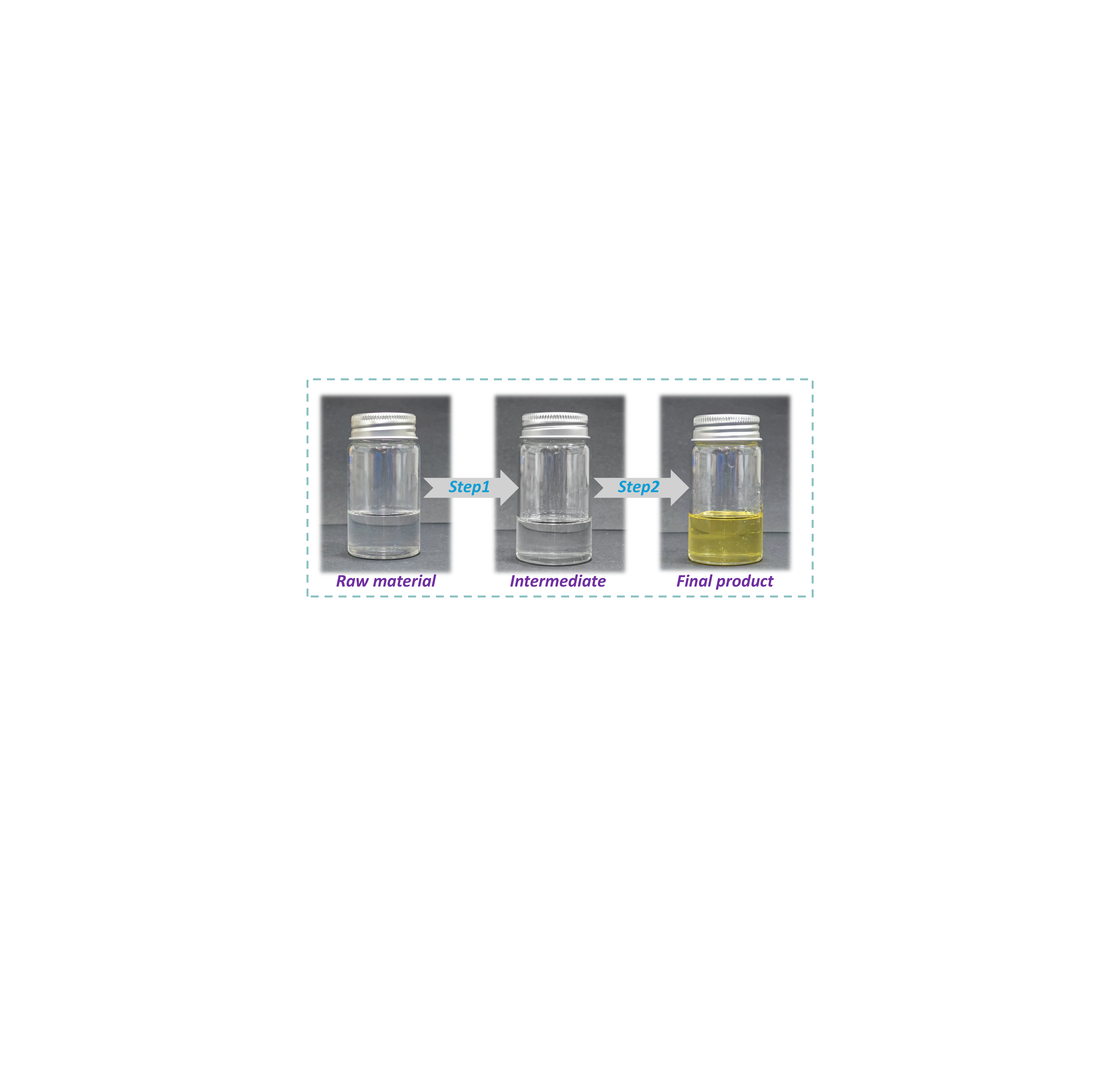


**Figure S3. Appearance of the reactants and products before and after the two-step reaction.**

**
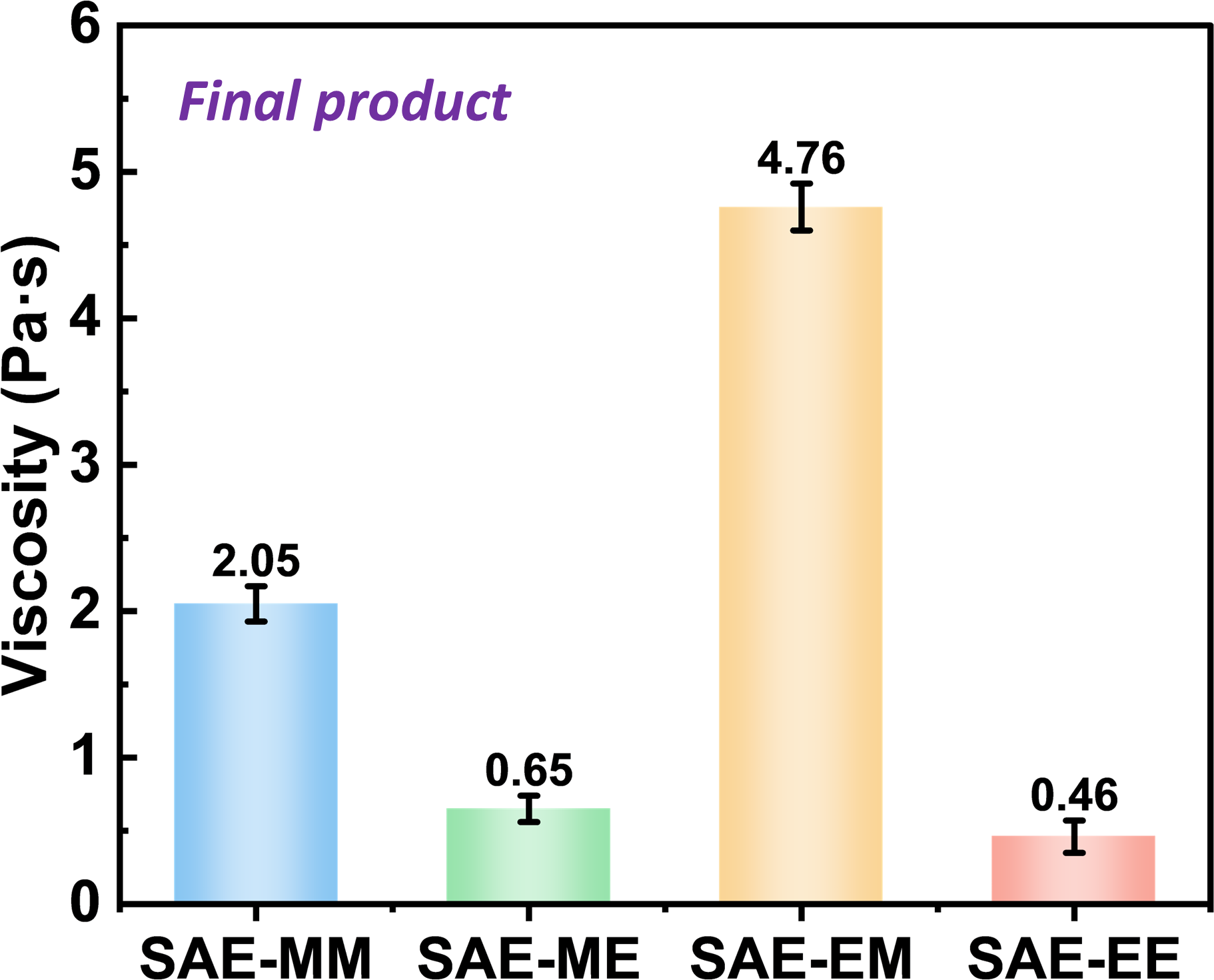
**

**Figure. S4. Viscosities of SAEs synthesized from different reactants.**

**
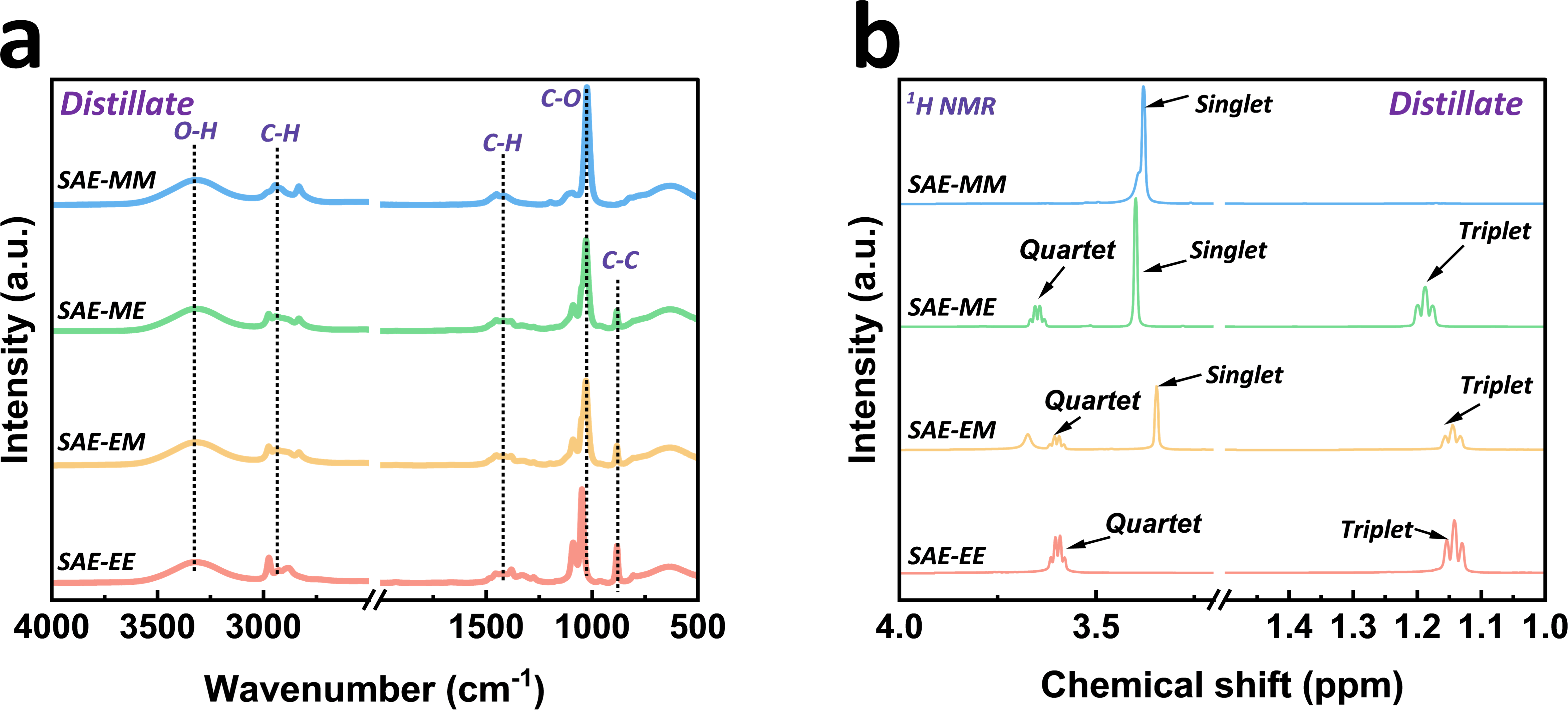
**

**Figure S5. (a)** FT-IR spectra and **(b)** ^1^H NMR spectra for the collected distillates from the syntheses of different SAEs.

The weak resonance at ~3.7 ppm in the SAE-EM distillate is attributed to trace methoxy-containing silane species (e.g., residual EPTMS) co-distilled during the dealcoholization process.


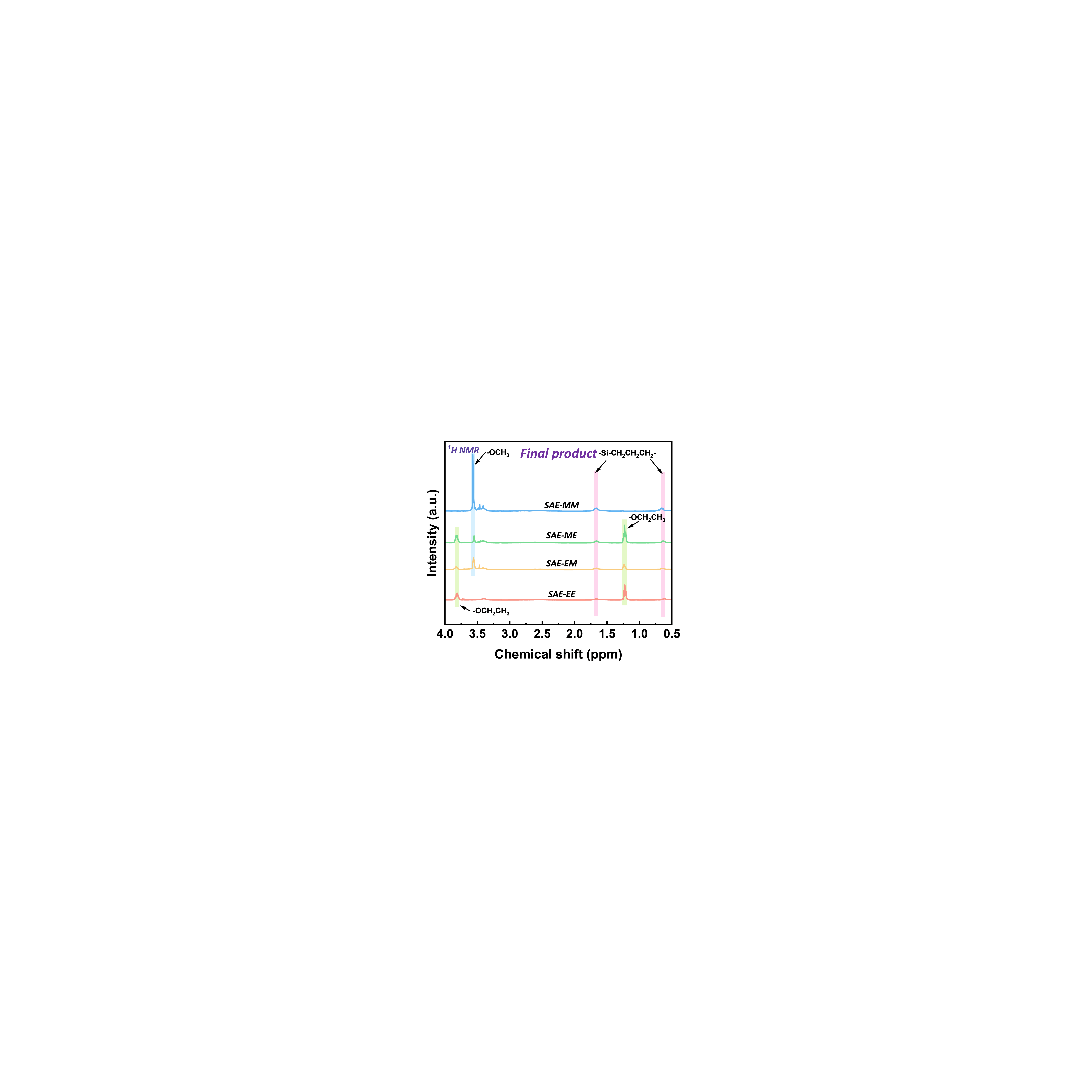


**Figure S6. ^1^H NMR spectra of SAEs.**

**
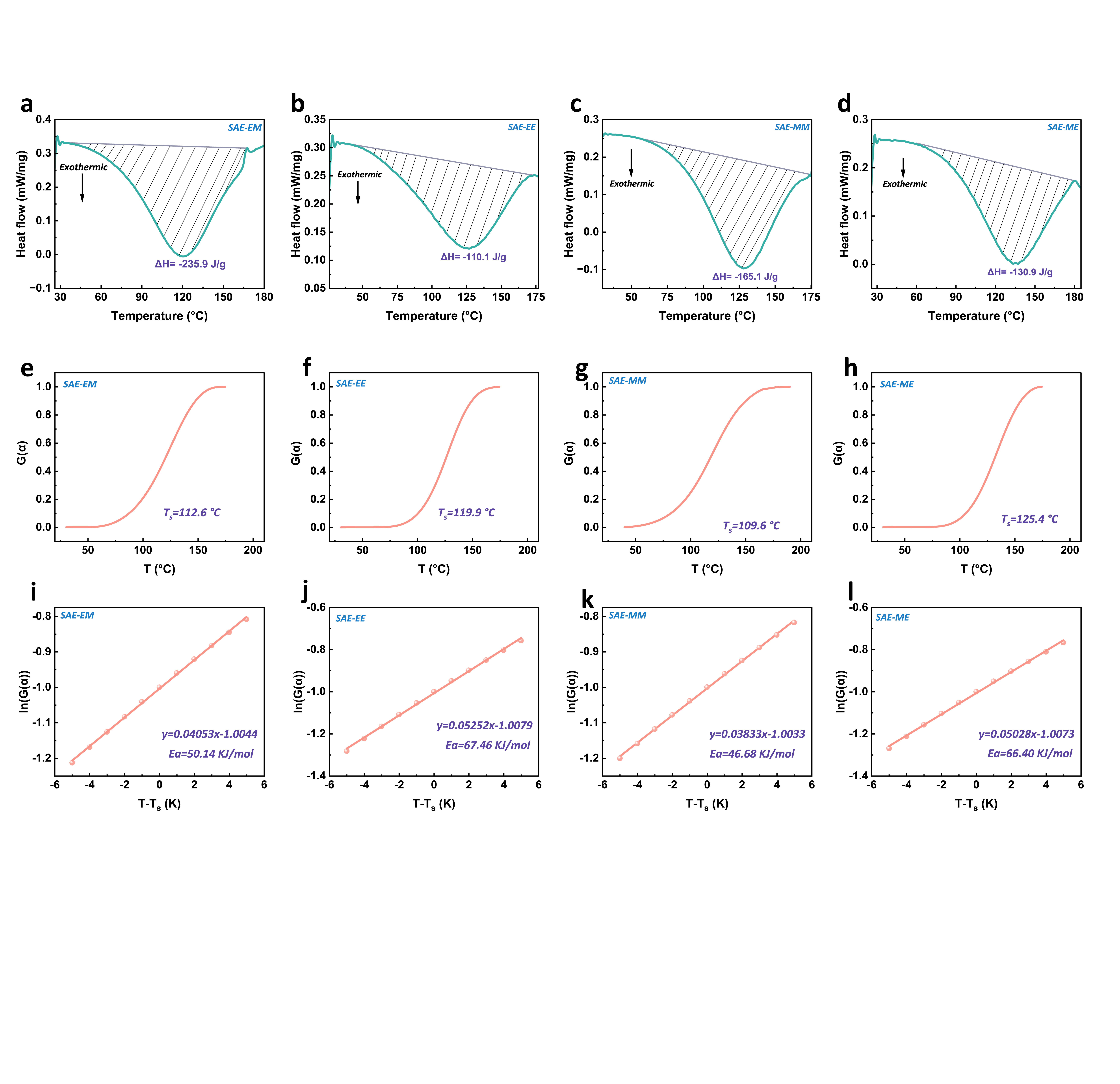
**

**Figure S7. Detailed kinetic analysis of the four SAE precursors (SAE-EM, -EE, -MM, and -ME). (a-d)** Non-isothermal DSC curves (10 K/min) for each precursor, showing the exothermic curing peak and the corresponding integrated reaction enthalpy (ΔH). **(e-h)** Corresponding fractional conversion curves ($G\left( \alpha\right)$ vs. Temperature) derived from the integration of the DSC data, with the characteristic temperature (T_s_) marked for each precursor. **(i-l)** Horowitz-Metzger (HM) plots of $\ln\left( G\left( \alpha\right) \right)$ versus $\theta$ (where $\theta$ = T - T_s_) for each precursor, showing the linear regression fit. The apparent activation energy ($E_{a}$) was calculated from the slope of this line.


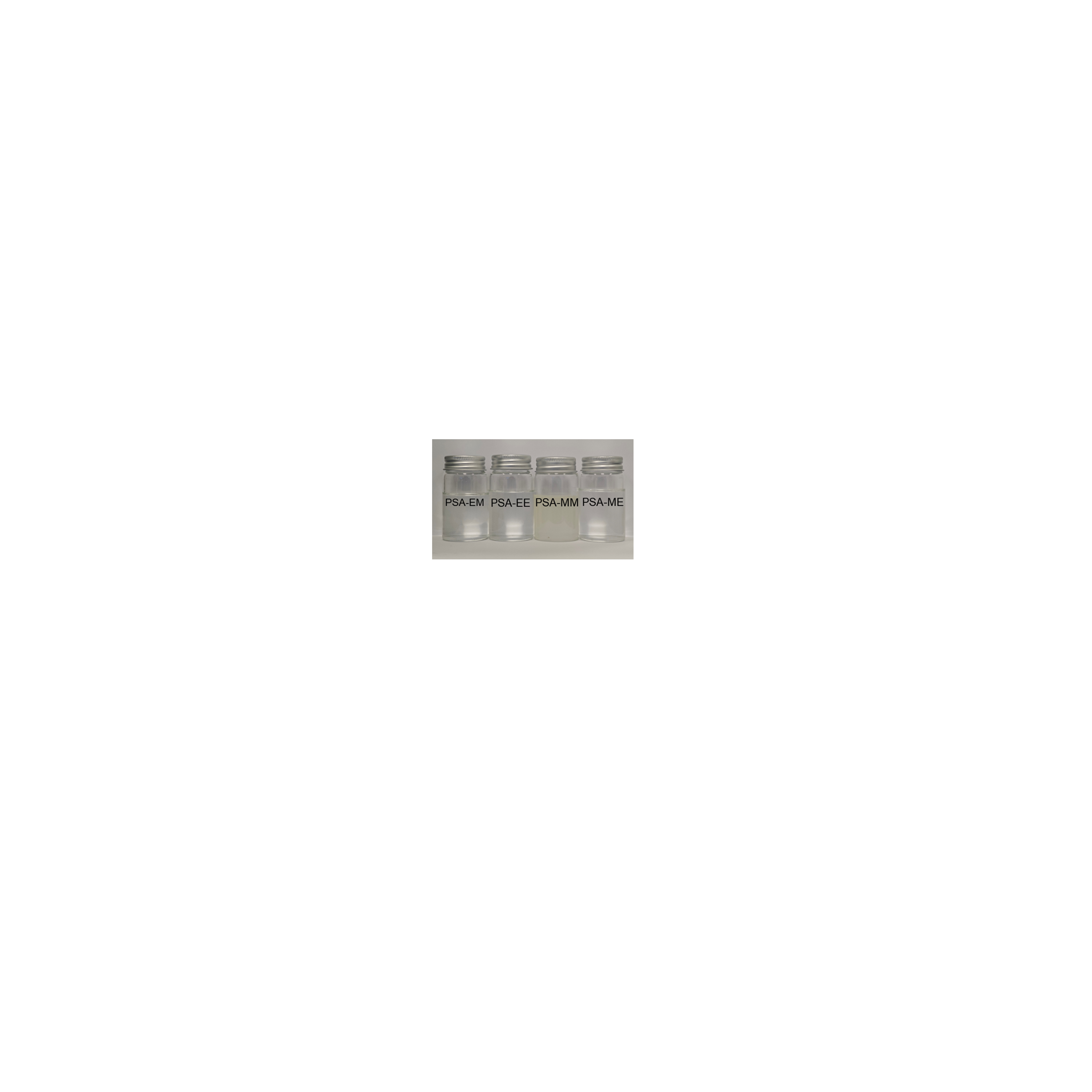


**Figure S8. Appearance of the pre-hydrolyzed solution after being left for 12 hours at 25 °C.**

**
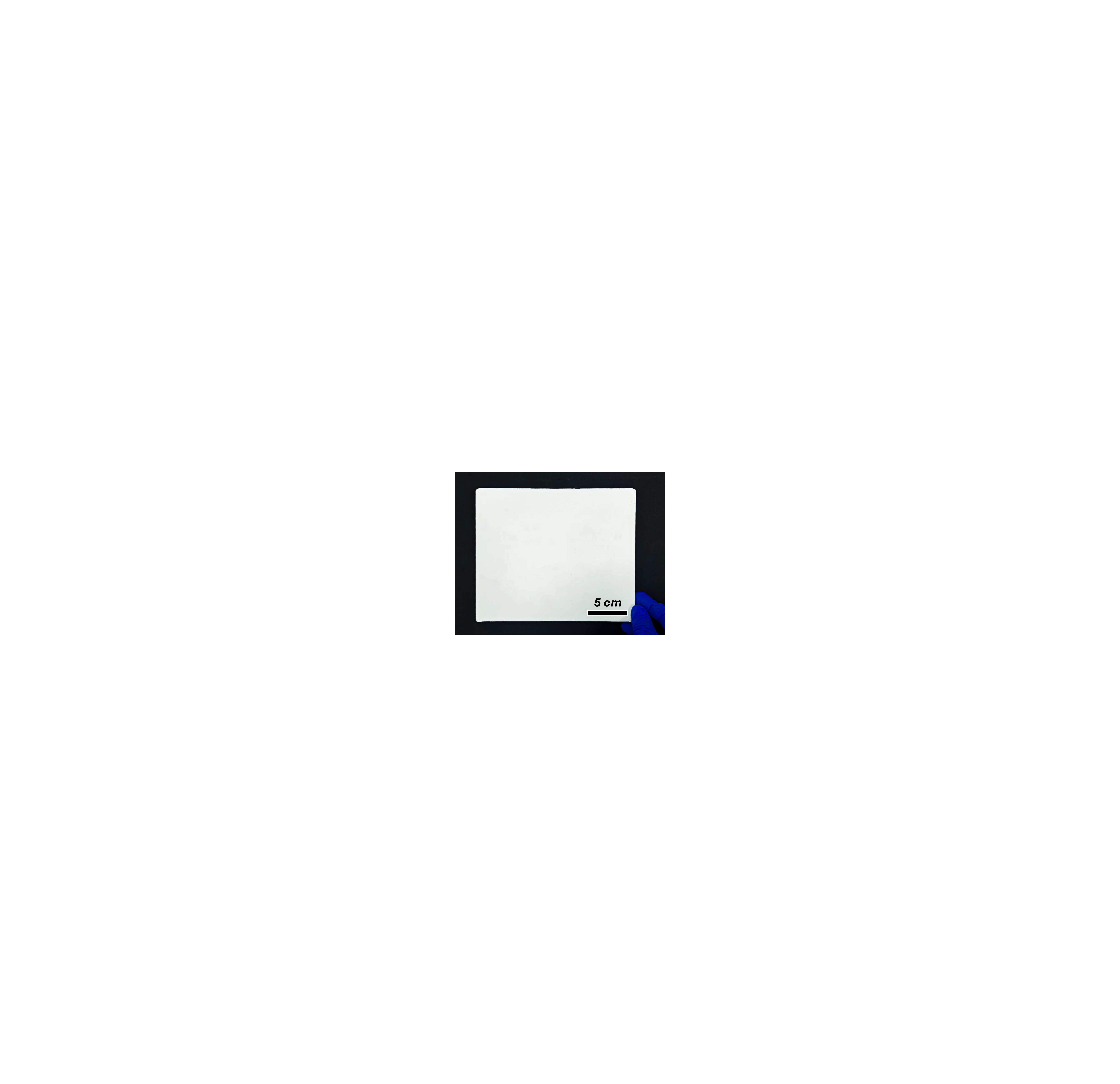
**

**Figure S9. Digital photograph of PSA-EM.**


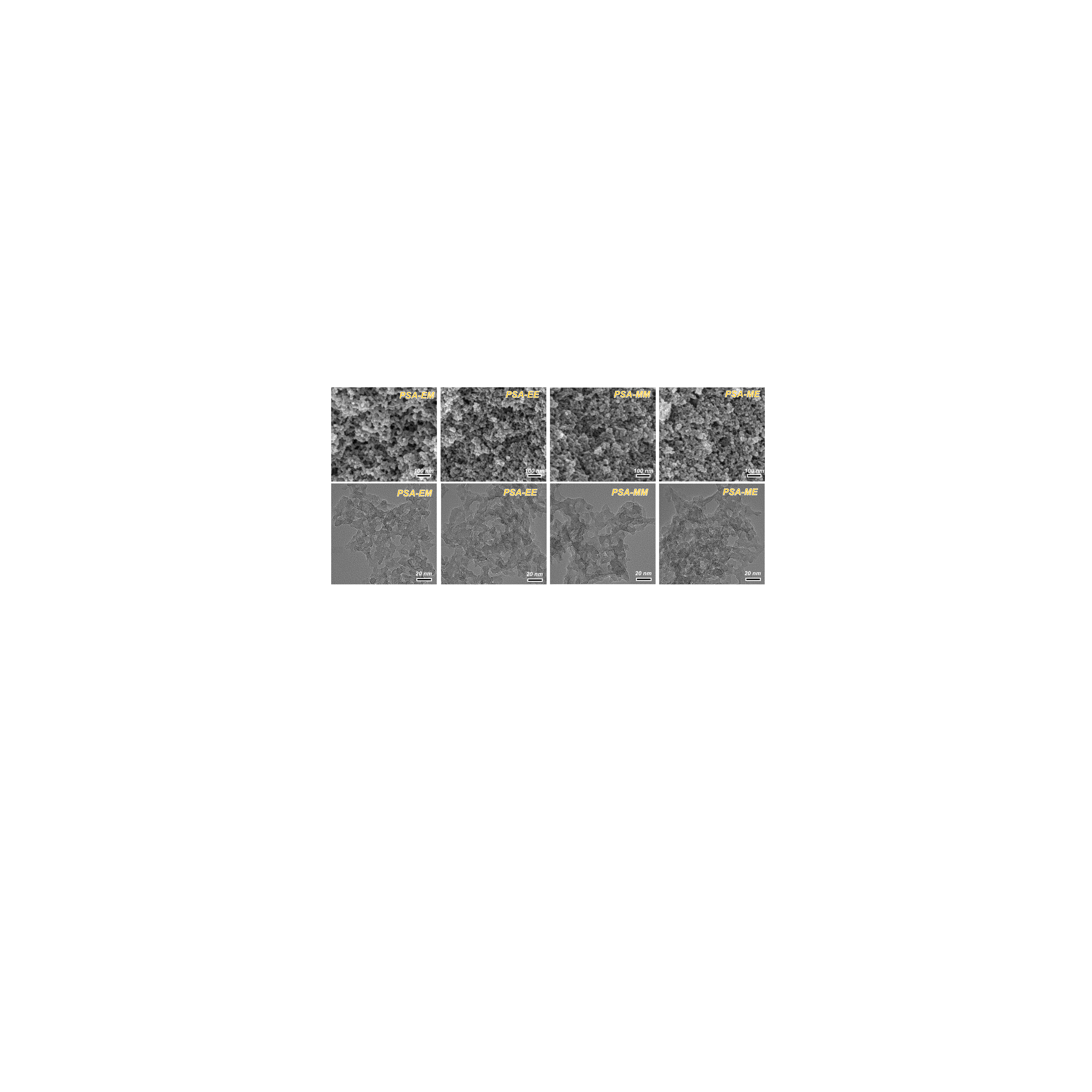


**Figure S10. SEM and TEM images of PSAs.**


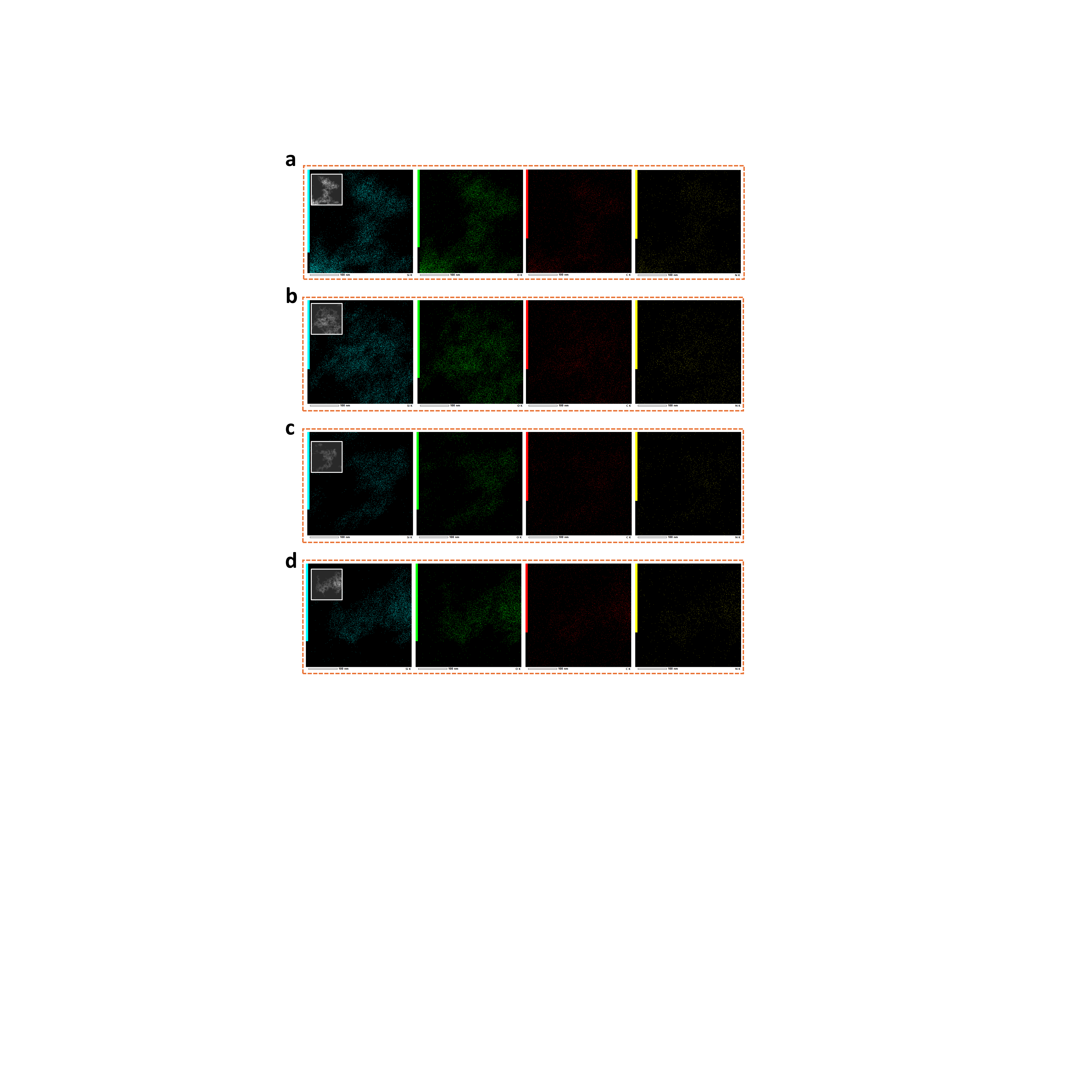


**Figure S11. Elemental mapping images of PSAs. a** PSA-EM**, b** PSA-EE**, c** PSA-MM**, and d** PSA-ME**.**


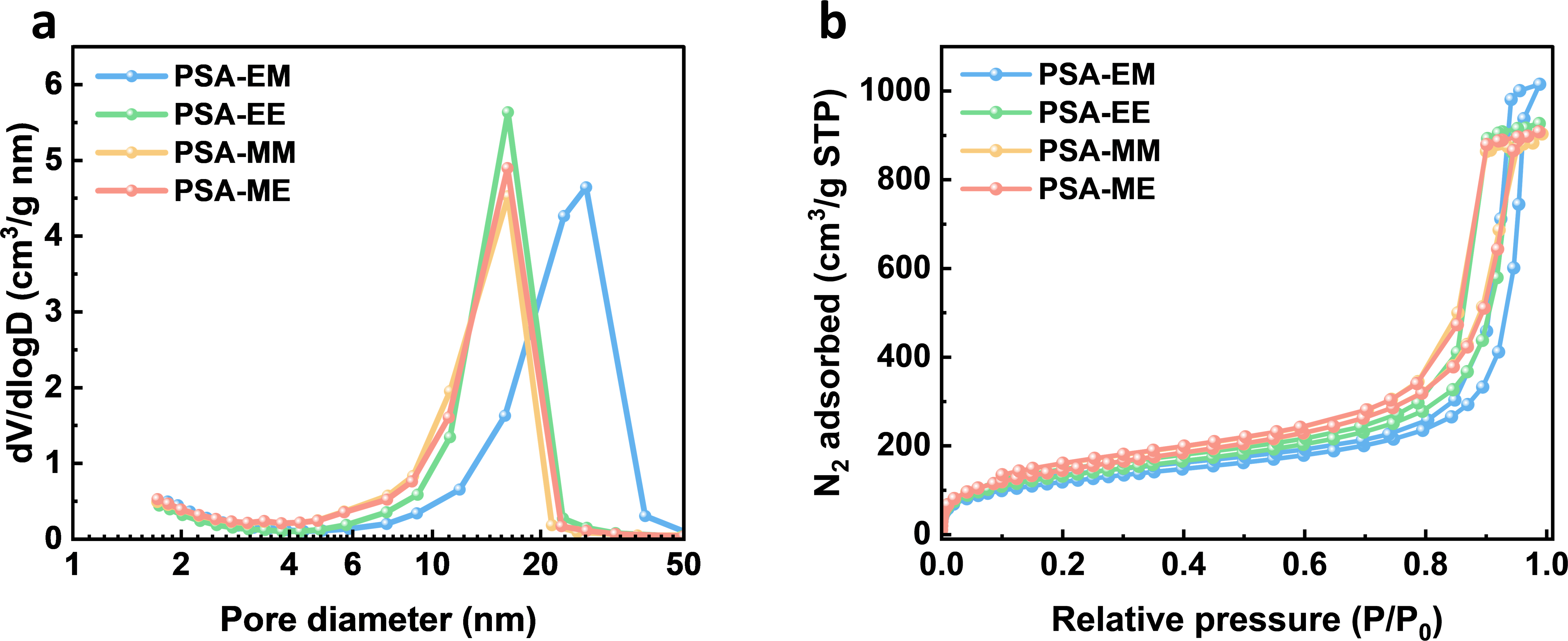


**Figure S12. BET Analysis Results of the PSAs**. **a** Pore size distribution of PSAs. **b** N_2_ adsorption/desorption isotherms of PSAs.


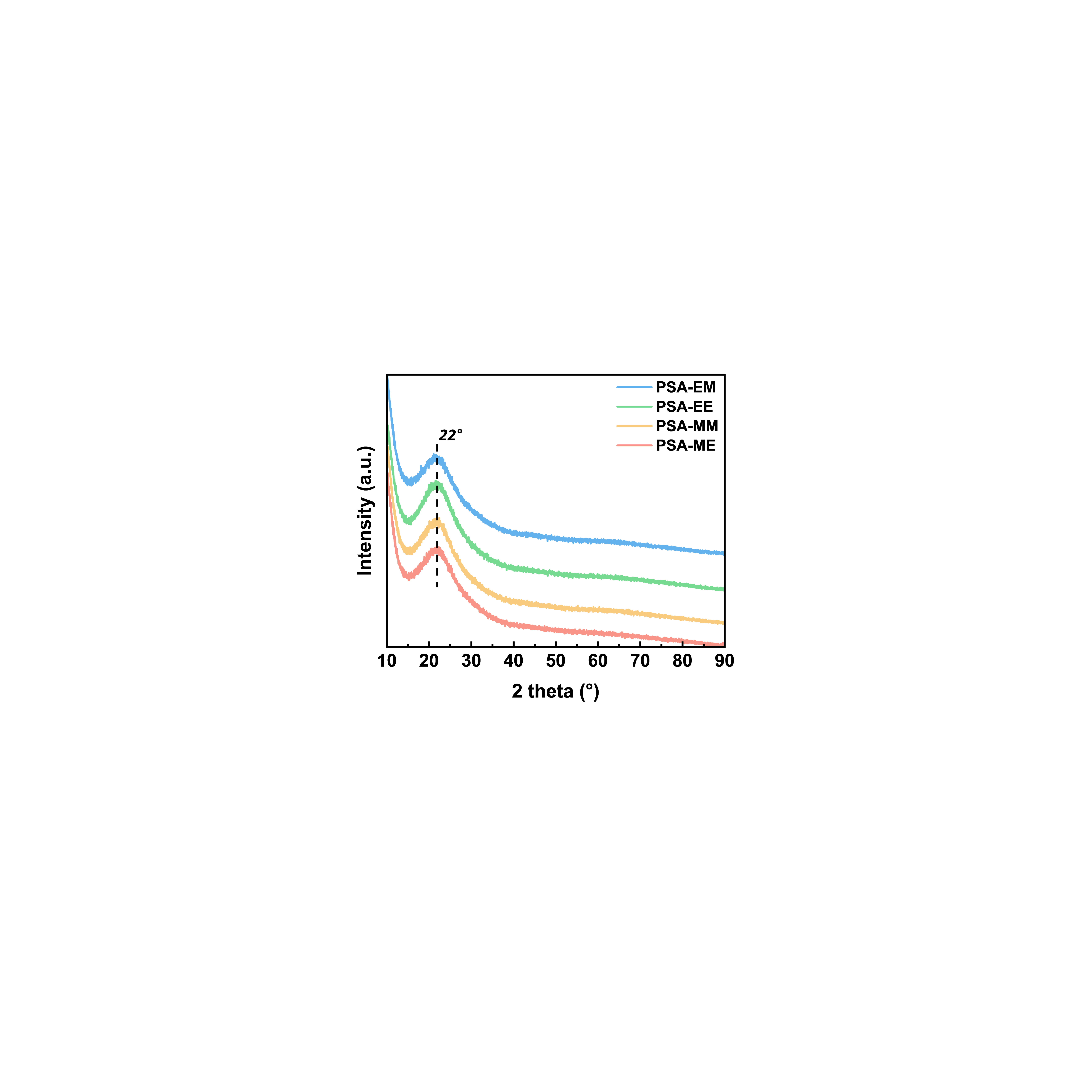


**Figure S13. X-ray diffraction (XRD) patterns of PSAs.**


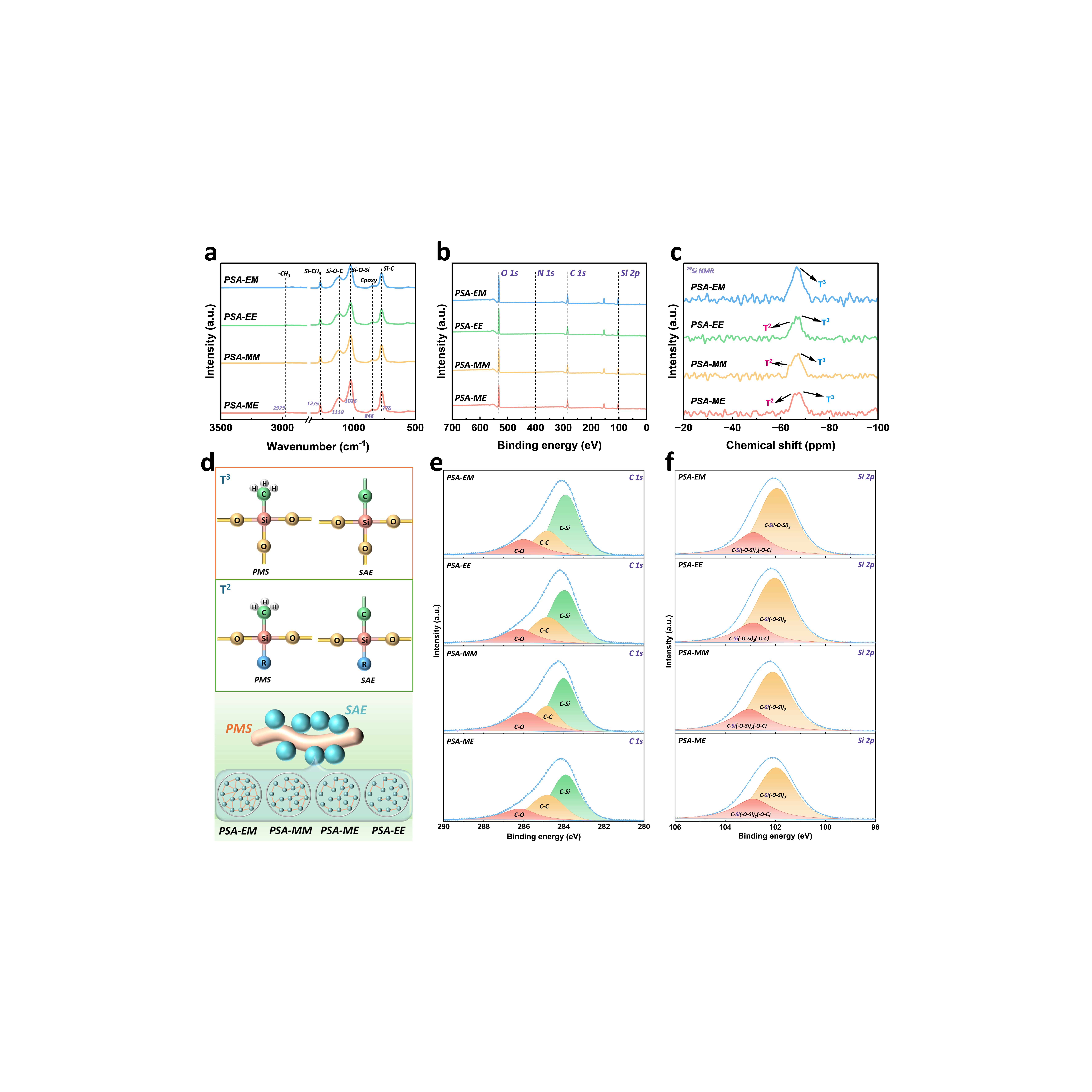


**Figure S14. Chemical structure of PSAs**. **a** FT-IR spectra of PSAs. **b** XPS survey scans of PSAs. **c** solid-state ^29^Si NMR spectra of PSAs. **d** Schematic diagram of the chemical structure. **e, f** C 1s (**e**) and Si 2p (**f**) XPS spectra of PSAs.

The chemical structures of the PSAs are investigated using X-ray diffraction (XRD), Fourier-transform infrared (FT-IR) spectroscopy, and nuclear magnetic resonance (NMR), with the results presented in Figure S13 and 14. The XRD patterns for all samples (Figure S13) display a single, broad diffraction peak centered at approximately 2θ=22°, which is characteristic of an amorphous silica structure and indicates the presence of an extensive Si-O-Si network.^[7]^ This siloxane network is further confirmed in the FT-IR spectra (Figure S14a) by the intense, sharp absorption peak at 1026 cm^−1^. Additionally, the absorption peaks at 2975 cm^−1^ and 1275 cm^−1^ correspond to the -CH_3_ and Si-CH_3_ groups from the PMS precursor, respectively.^[8,9]^ Notably, the characteristic peak at 846 cm^−1^, which corresponds to unreacted epoxy groups in the SAE precursor, essentially disappears in the final PSA. This indicates that the epoxy groups undergo a thermally induced ring-opening and participate in further intermolecular crosslinking reactions during the high-temperature aging process at 150°C. This additional crosslinking further enhances the mechanical properties of PSAs. The detailed mechanism for this reaction is discussed in previous work.^[10]^

The chemical structure of the silicon atoms is investigated using ^29^Si NMR spectroscopy. As shown in Figure S14b and c, all the samples are predominantly composed of doubly-bridged (T^2^, approx. -55 to -65 ppm) and triply-bridged (T^3^, approx. -65 to -75 ppm) structural units, though the relative proportion of T^2^ and T^3^ units varies significantly.^[11,12]^ Notably, the spectrum for PSA-EM displays a sharp, symmetric peak exclusively in the chemical shift region assigned to T^3^ units, which indicates that its solid-state network possesses an exceptionally high degree of condensation and structural integrity. In contrast, the spectra for PSA-MM, PSA-ME, and PSA-EE all exhibit a distinct shoulder peak corresponding to T^2^ structures, revealing the presence of numerous incompletely condensed units and structural defects.^[13]^ This microstructural difference provides strong corroboration for the previously proposed kinetic hypothesis: the differential reactivity of the alkoxy groups in the PSA-EM precursor (SAE-EM) is believed to facilitate a more orderly polymerization, leading not only to a more viscous liquid precursor but also to a final cured aerogel network with more complete crosslinking and fewer defects.


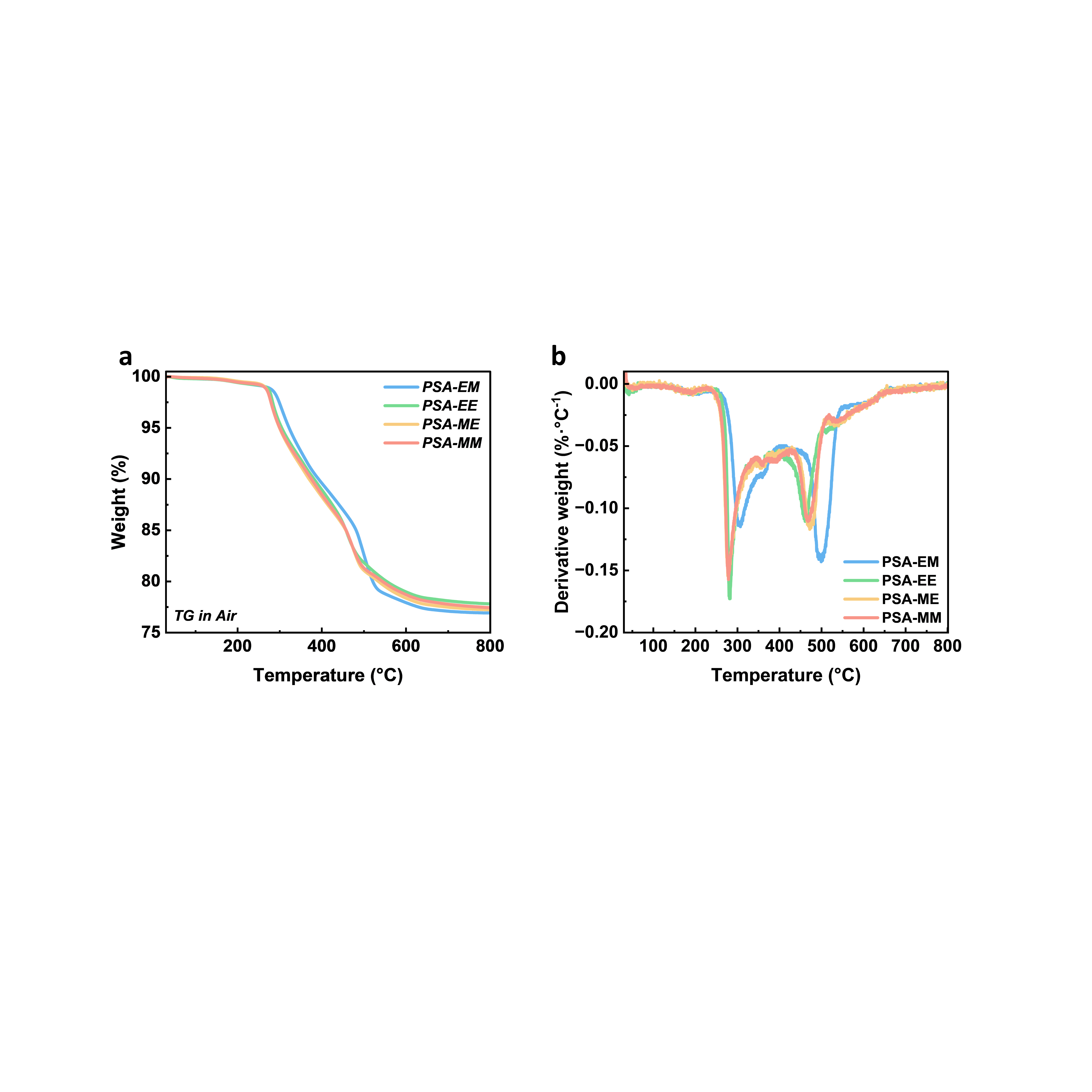


**Figure S15. TG and DTG curves of the PSAs in air. a** TG curves. **b** DTG curves.


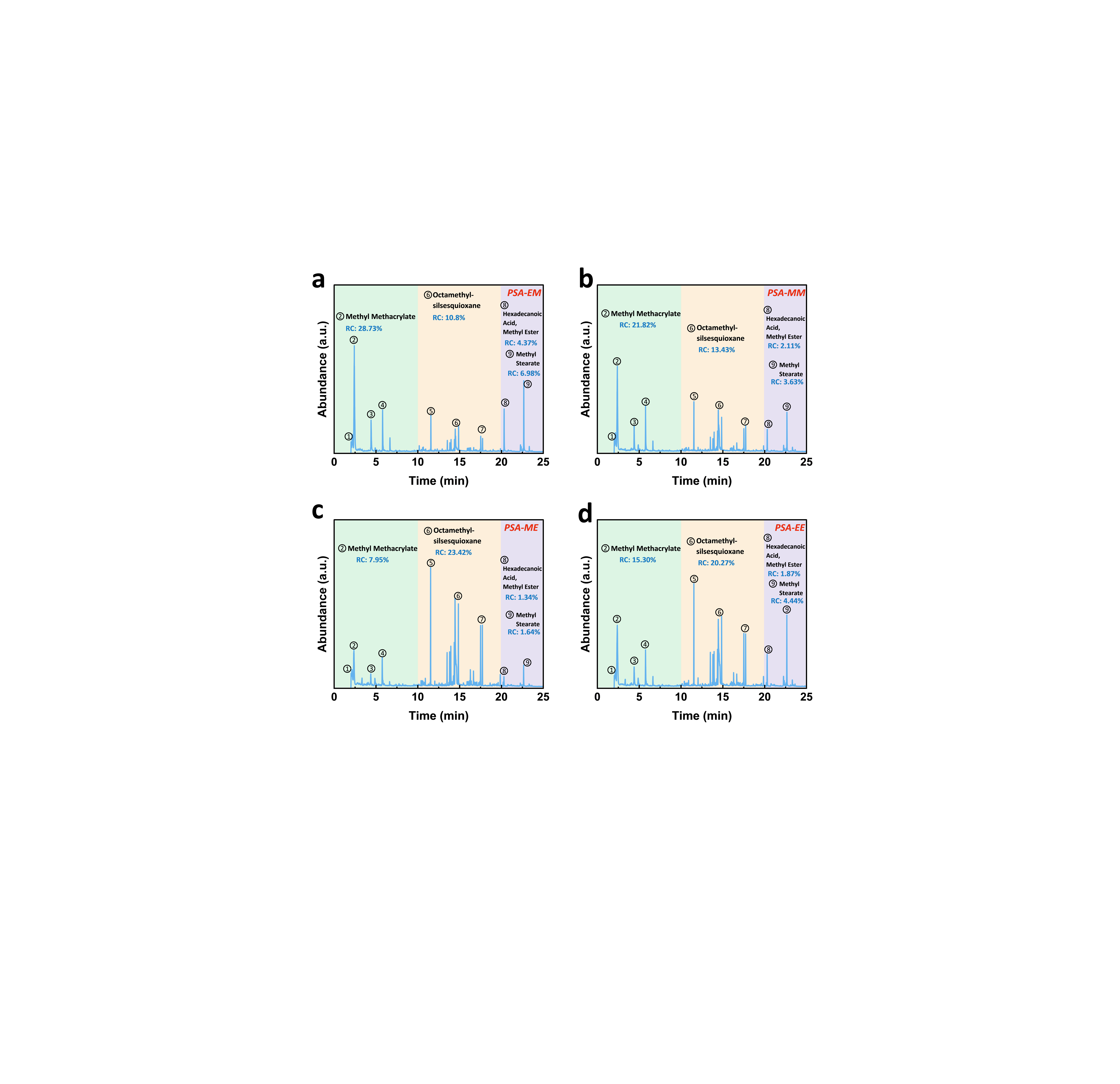


**Figure S16.** GC/MS chromatogram of PSAs: (**a**) PSA-EM, (**b**) PSA-MM, (**c**) PSA-ME, and (**d**) PSA-EE.


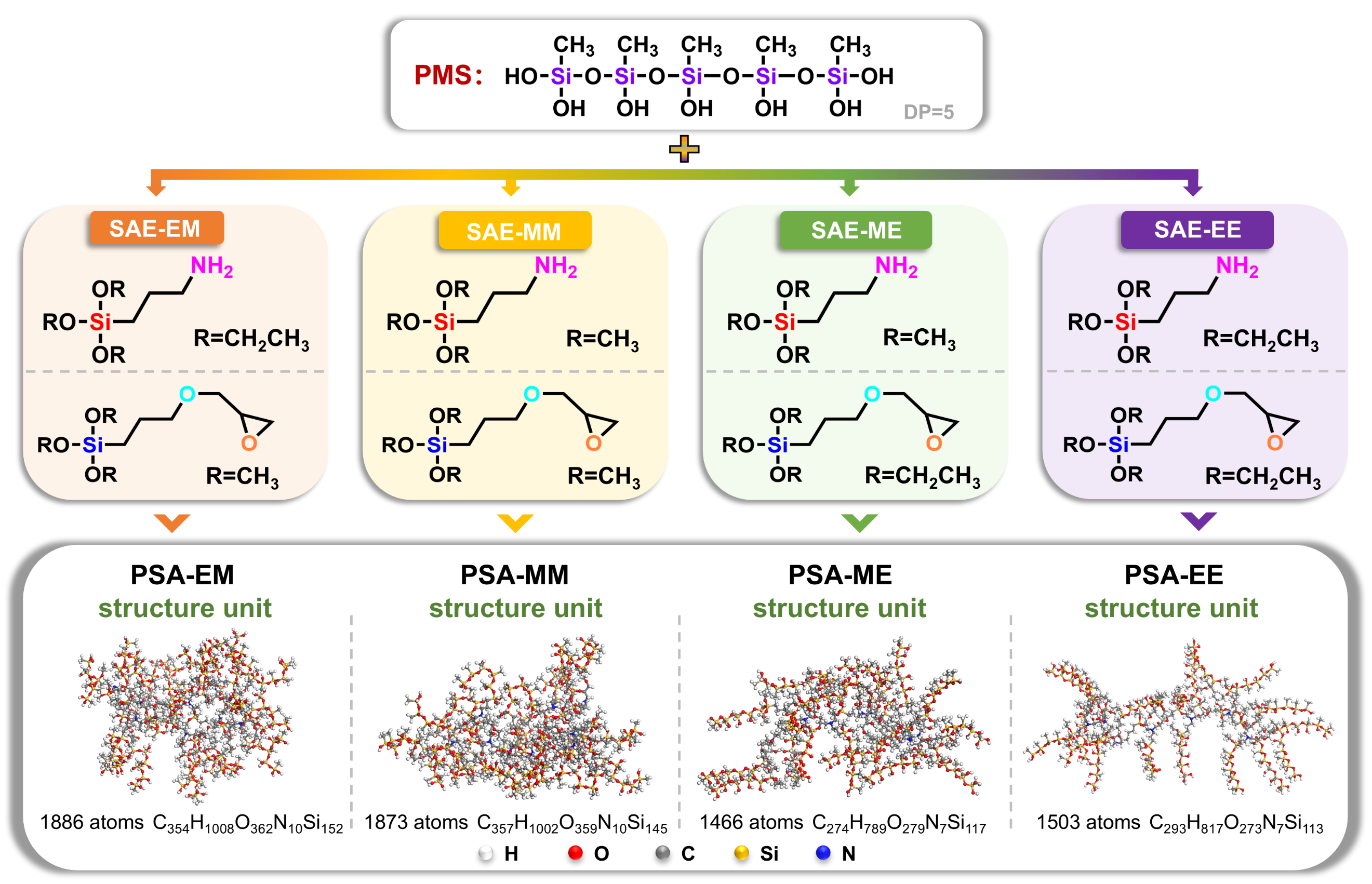


**Figure S17. Construction Pathway of Different PSAs Structural Units.**

Based on the chemically synthesized structures of the different SAEs proposed in the experiment (SAE-EM, SAE-MM, SAE-ME, SAE-EE), each was cross-linked with PMS of a polymerization degree of 5. This process resulted in the formation of four distinct PSA structural units with varying degrees of cross-linking (PSA-EM, PSA-MM, PSA-ME, PSA-EE). Their basic structures and chemical formulas are shown in Figure S17.


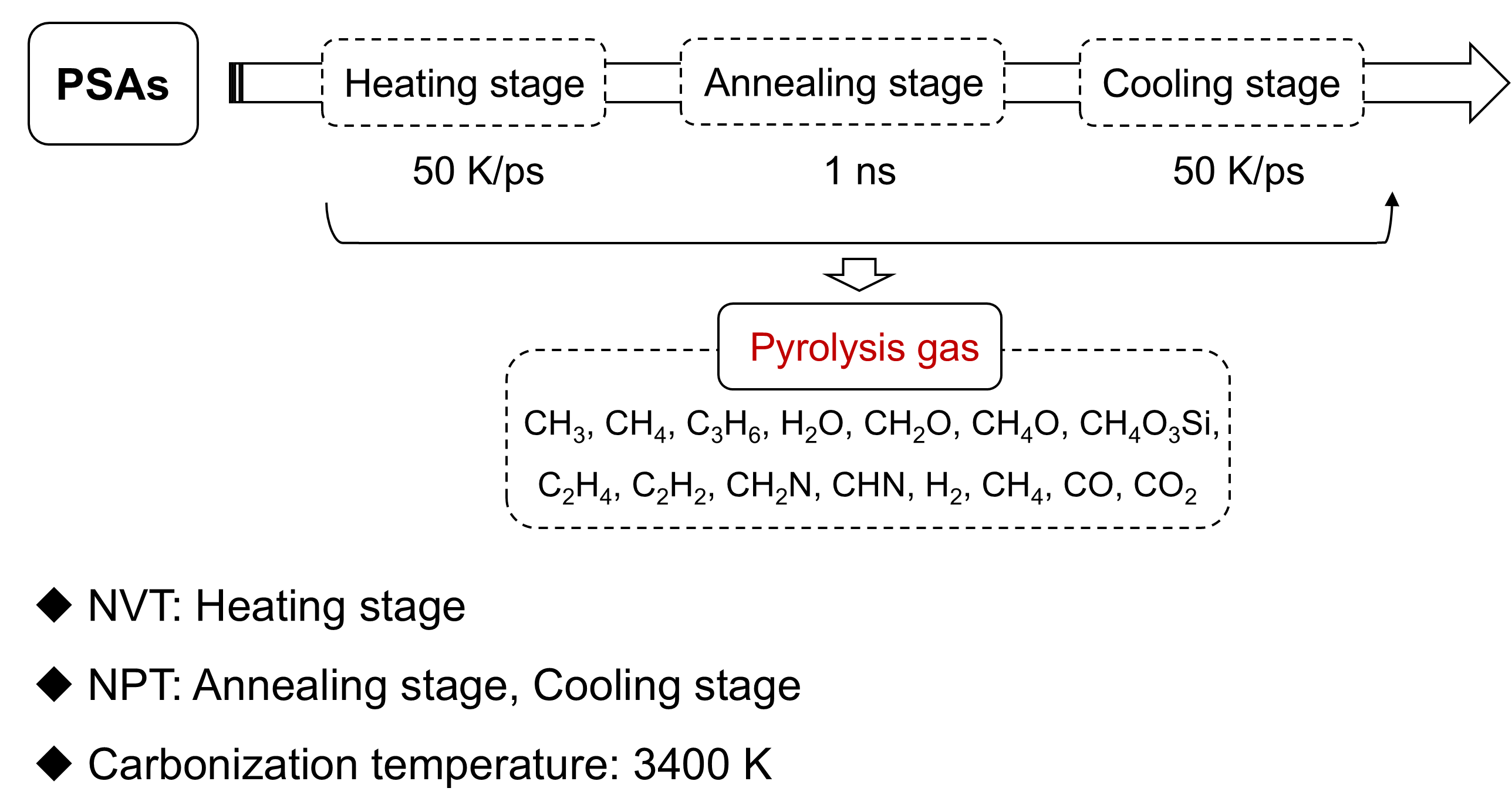


**Figure S18. Pyrolysis carbonization simulation process of PSAs.**

All pyrolysis and carbonization simulations were conducted using the Large-scale Atomic/Molecular Massively Parallel Simulator (LAMMPS) with the Velocity-Verlet time integration algorithm. The Qeq charge equilibration method was employed to calculate and update atomic charges. Notably, a Nosé-Hoover thermostat and barostat with damping coefficients of 100 fs and 1000 fs, respectively, were used to control the temperature and pressure of the simulation system. The simulations employed the CHONSSiNaP-tribology force field parameter set with a time step of 0.1 fs to model the continuous formation and breaking of chemical bonds in the PSAs systems.

Based on the different PSA models shown in Figure 4a, the system was rapidly heated to 3400 K at a rate of 50 K/ps. The results indicated that the types of pyrolysis products from different PSA systems were generally consistent, mainly including small gaseous molecules such as CH_3_, CH_4_, C_3_H_6_, H_2_O, CH_2_O, CH_4_O, CH_4_O_3_Si, C_2_H_4_, C_2_H_2_, CH_2_N, CHN, H_2_, CO, and CO_2_. The primary difference lies in the amounts of each component produced. As shown in Figure S16, the pyrolysis and carbonization processes of different PSAs were further simulated using a low-temperature liquid quenching method. This process encompasses three stages: heating, annealing, and cooling, ultimately yielding the carbonization products of each PSA system. To simulate the experimental conditions where volatile pyrolysis gases are removed in a nitrogen atmosphere at constant flow rate, major pyrolysis gases were periodically removed from the system every 0.5 ps during the simulation.


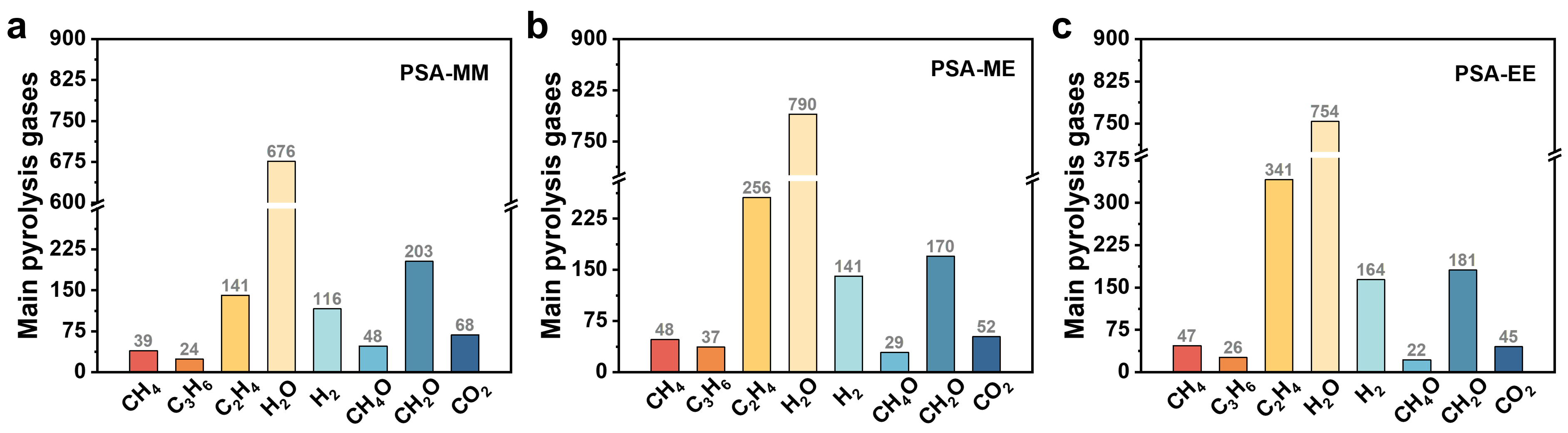


**Figure S19. Main types and numbers of pyrolysis gases of PSA-MM, PSA-ME, and PSA-EE.**


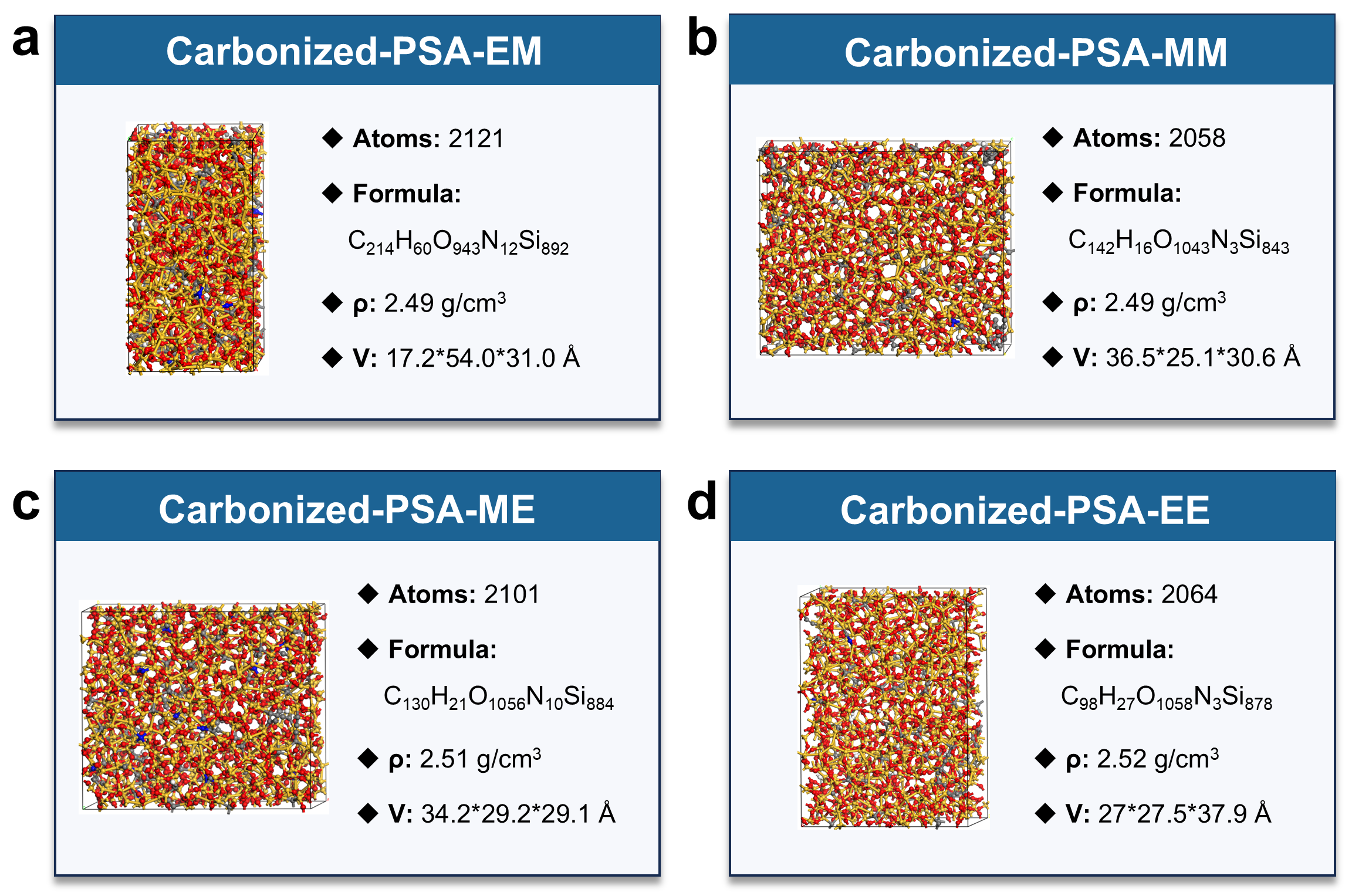


**Figure S20. Basic information of pyrolysis carbonization solid-phase products from Different PSAs.**


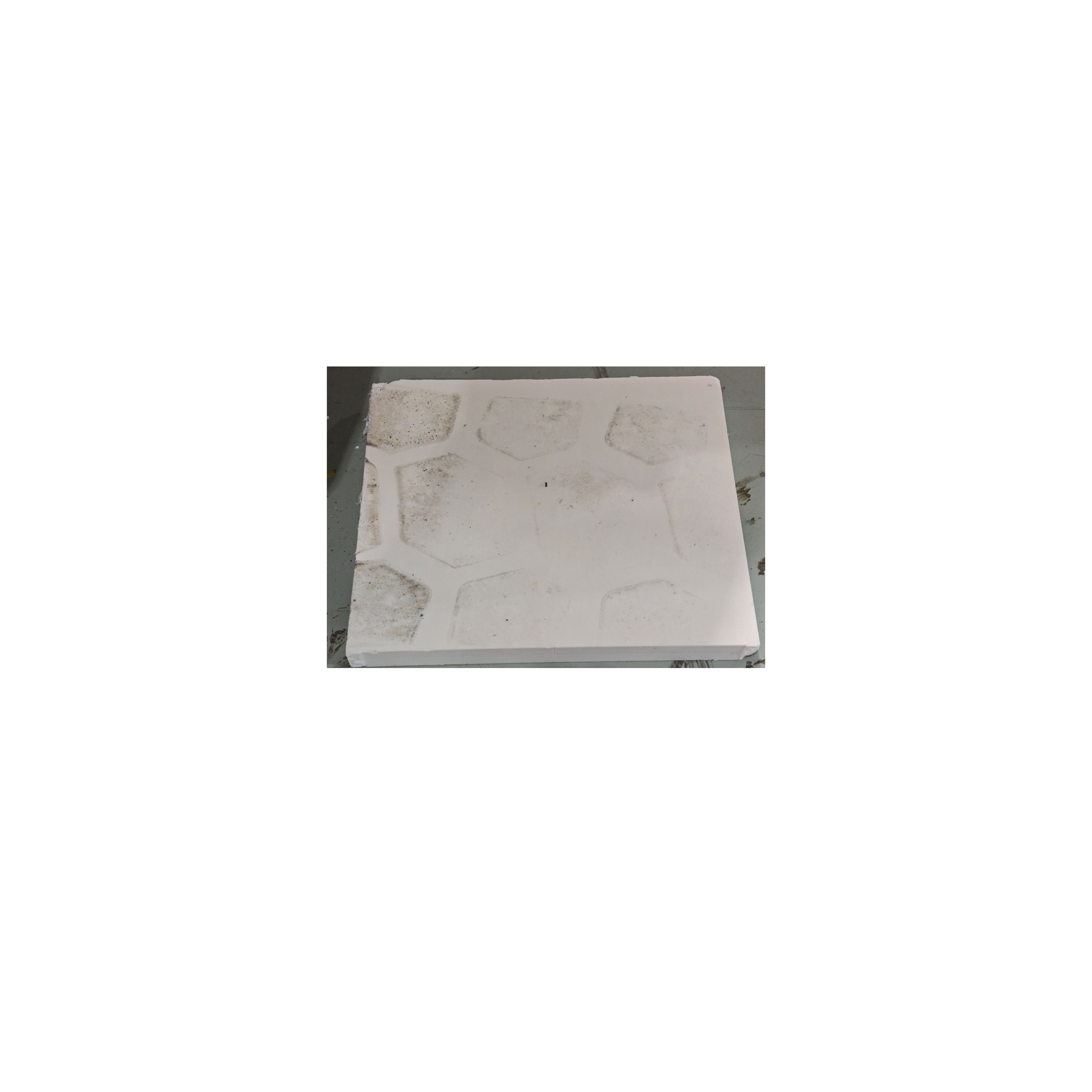


**Figure S21. PSA-EM after repeated compaction by forklift.**

**
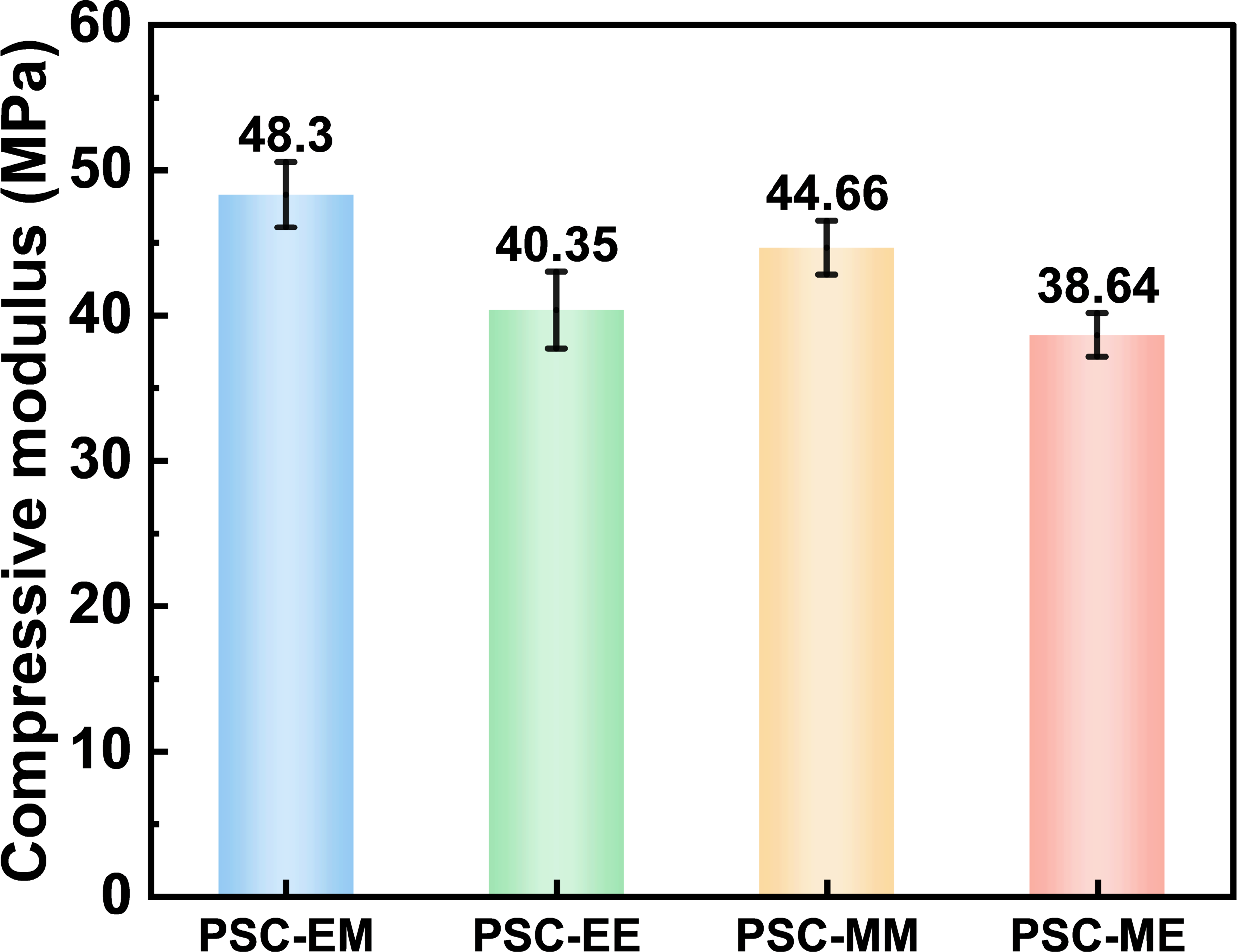
**

**Figure S22. Compression modulus of PSCs.**

**
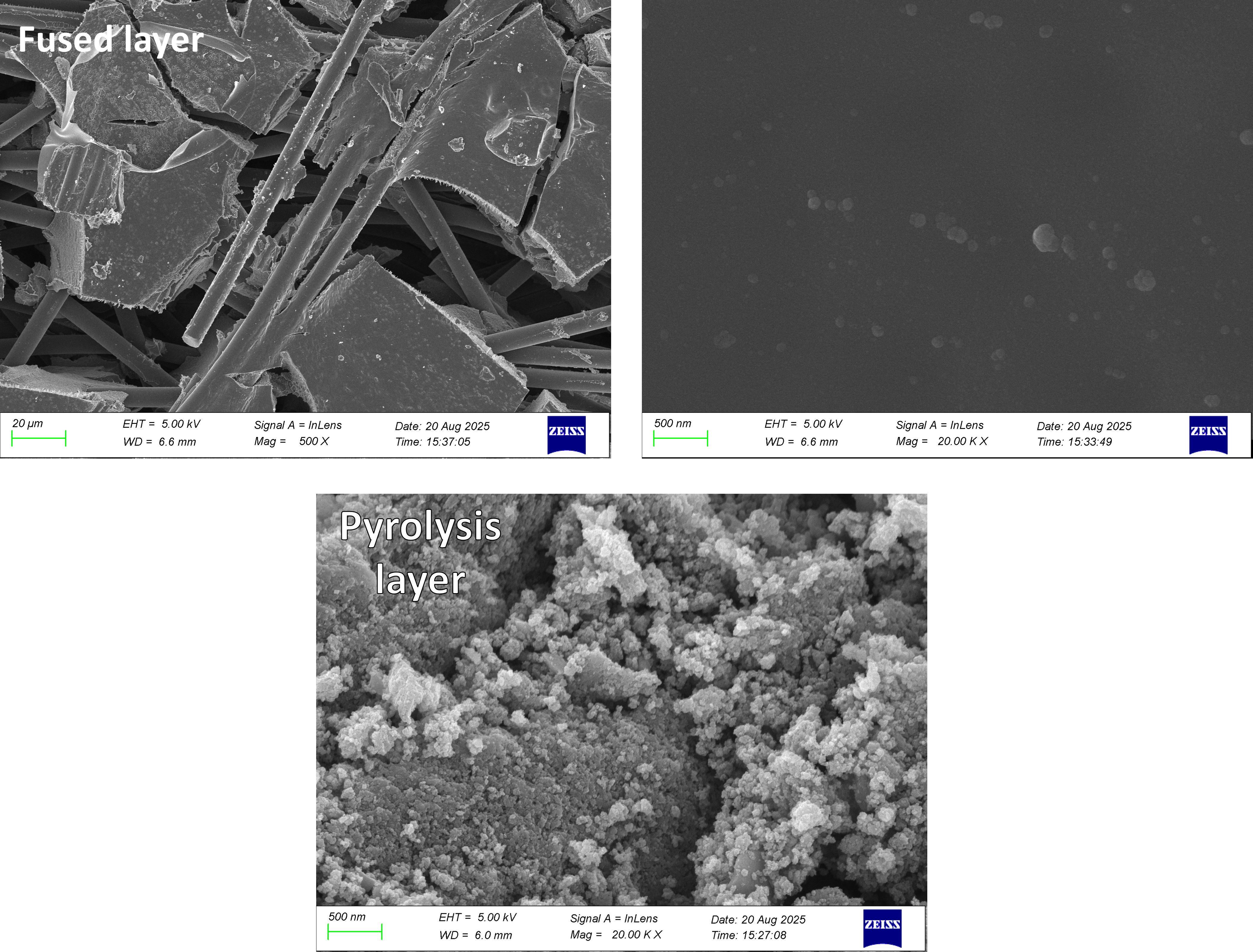
**

**Figure S23. SEM images of PSC-EE after test.**

**
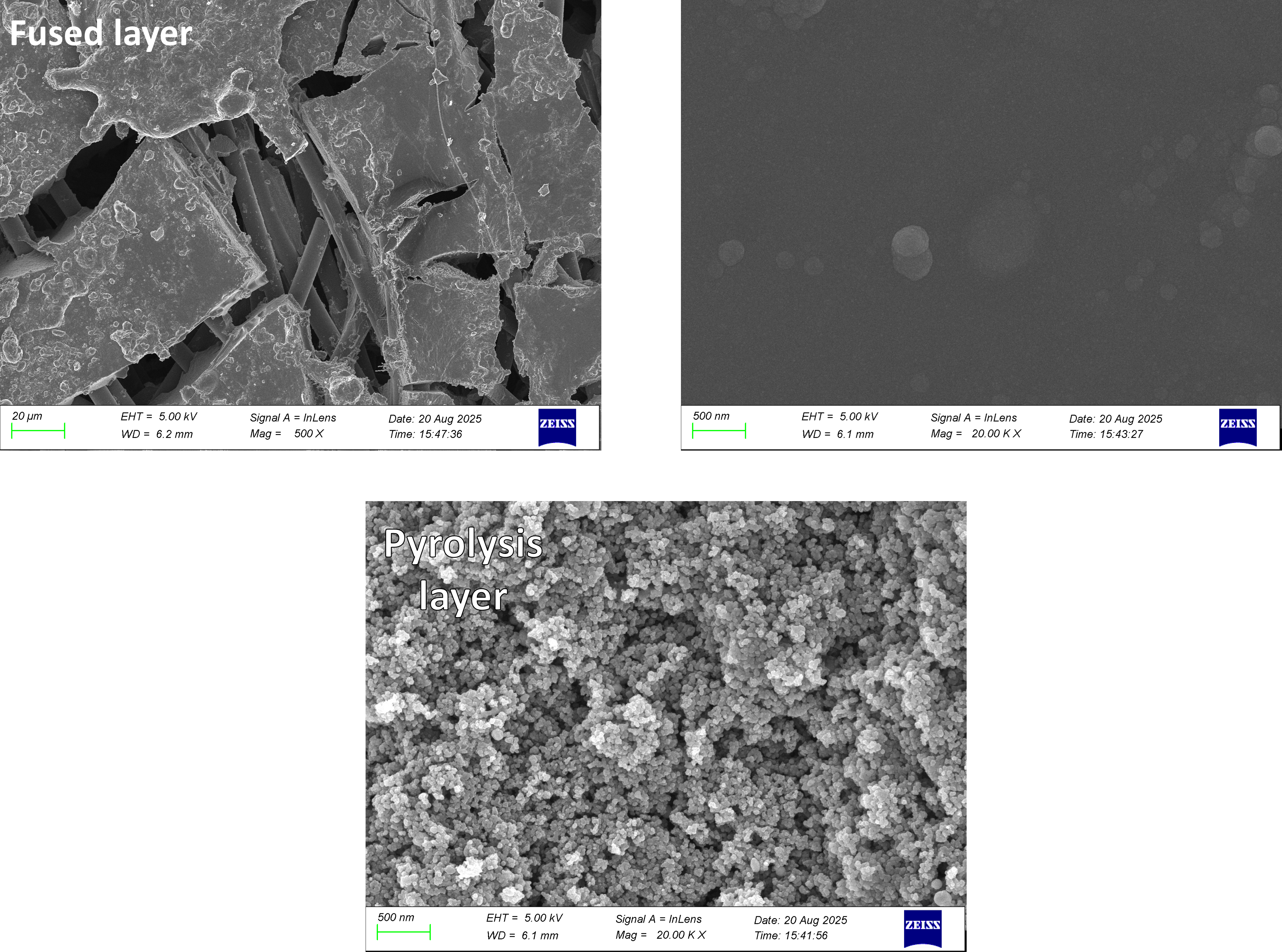
**

**Figure S24. SEM images of PSC-MM after test.**

**
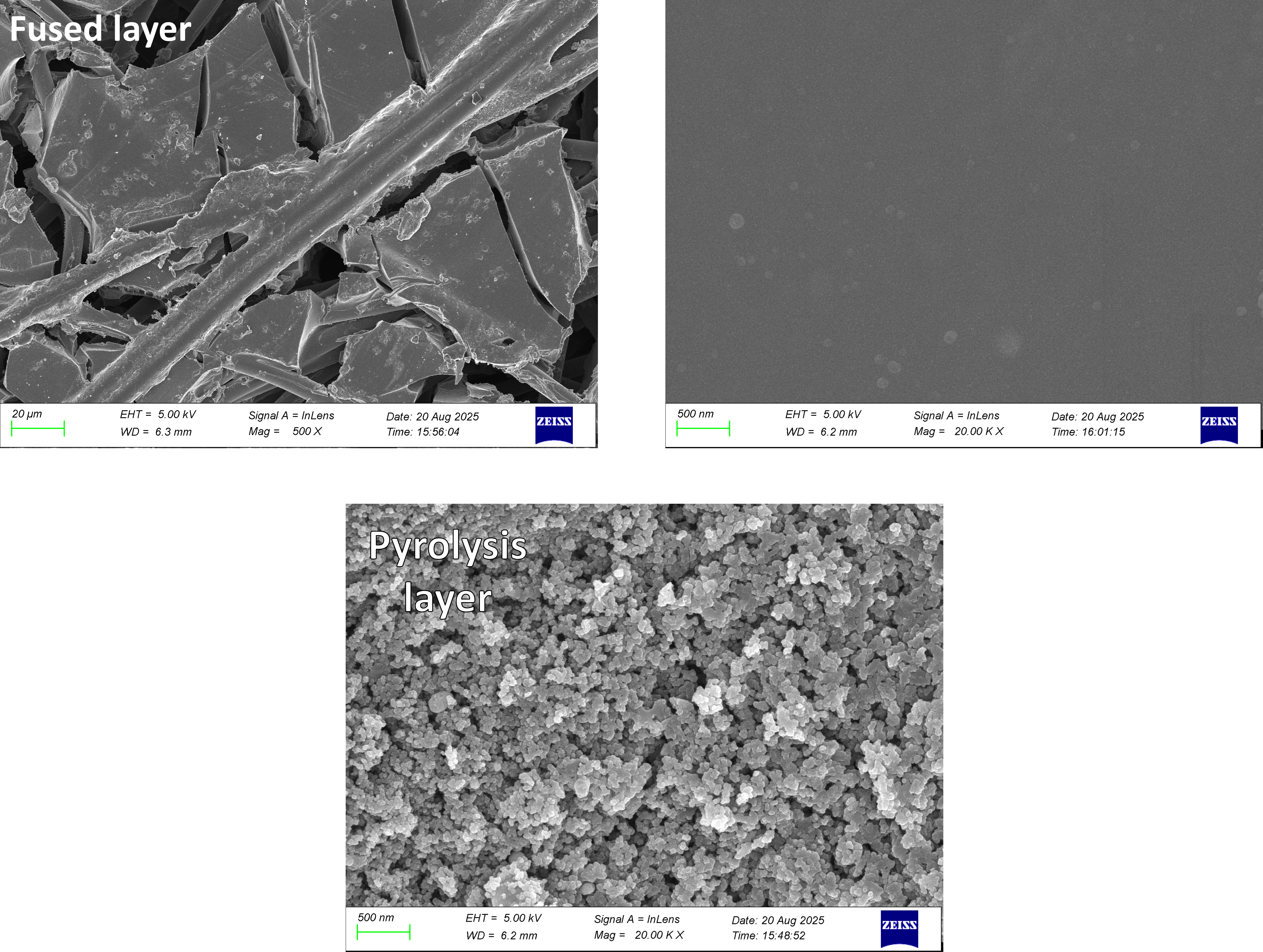
**

**Figure S25. SEM images of PSC-ME after test.**

**
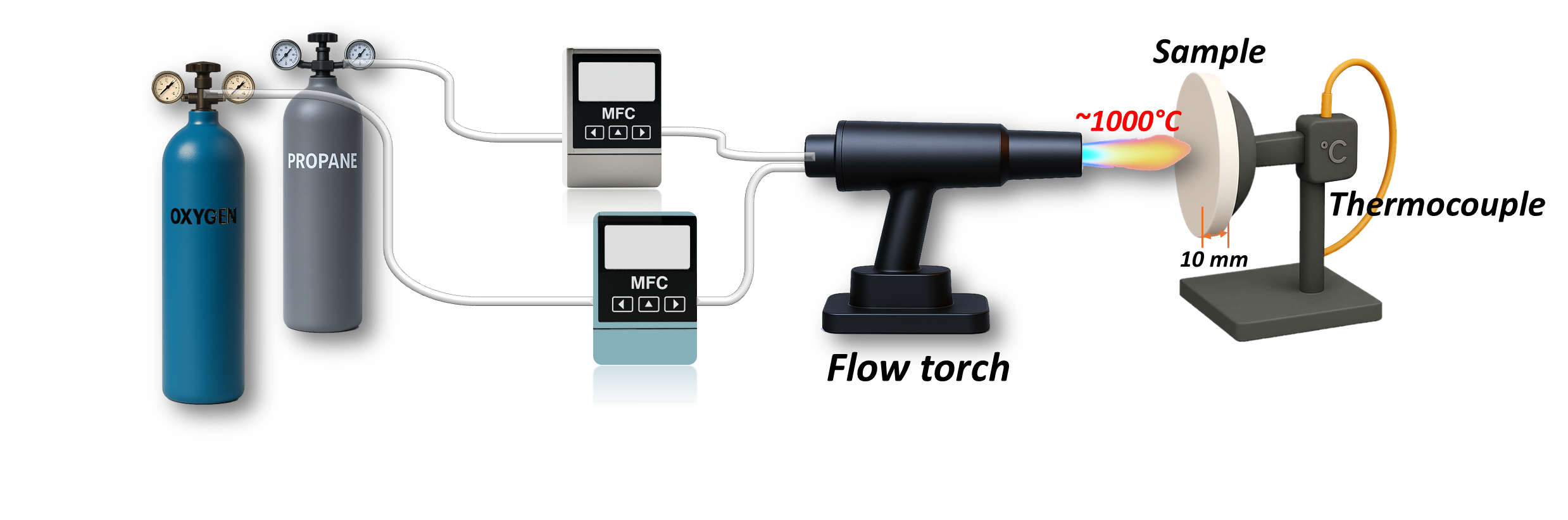
**

**Figure S26. Schematic diagram of oxy-propane ablation system.**


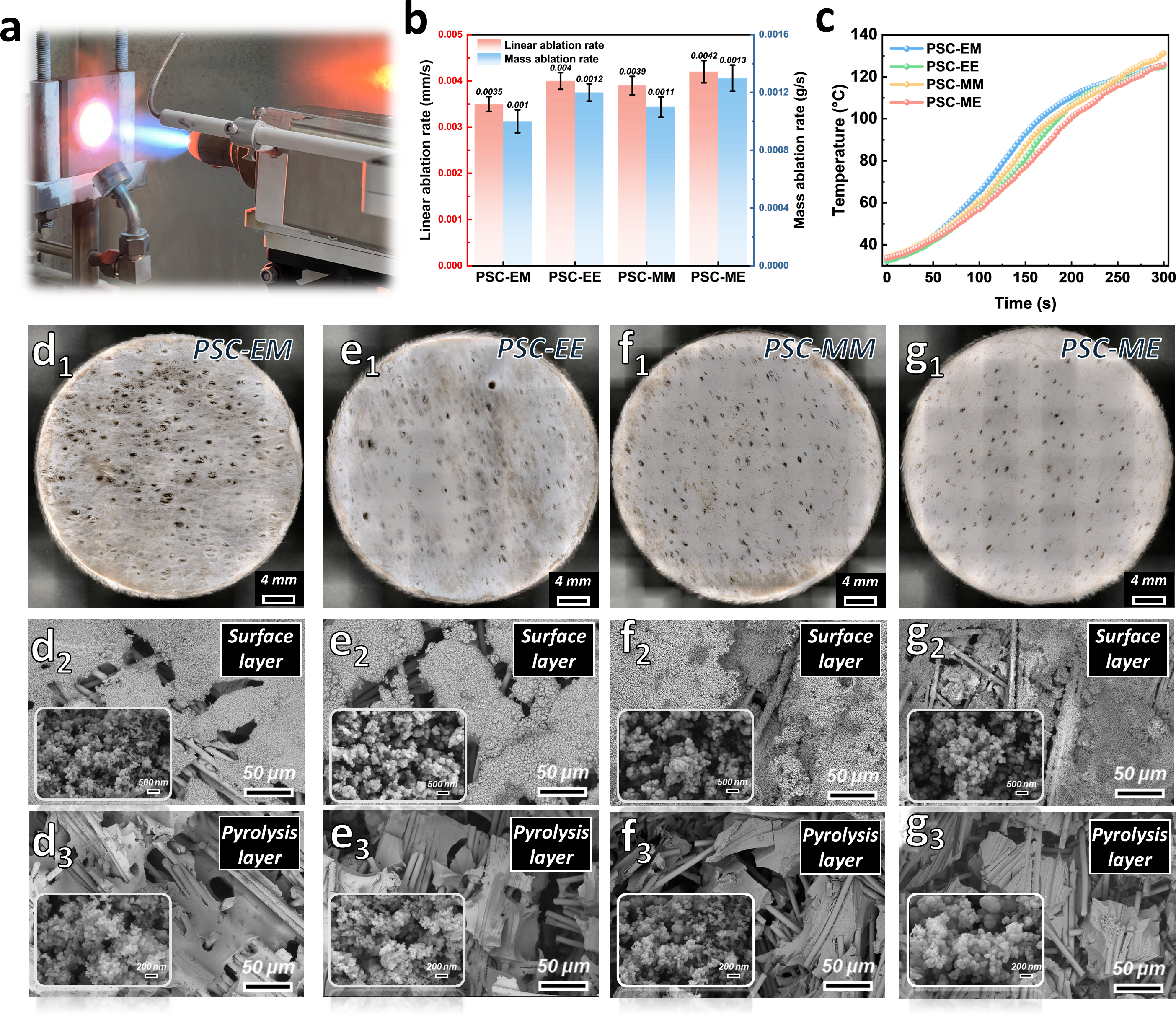


**Figure S27. Evaluation of the Ablation Resistance of PSCs. a** Photograph of ablation process. **b** The linear ablation rate (R_l_) and mass loss rate (R_m_) of PSCs under oxygen–propane flame with temperature of 1000 °C. **c** The backside temperature of PSCs under oxygen–propane flame with temperature of 1000 °C. **d_1_-g_3_** Surface macro and micro morphology of PSCs under an oxygen–propane flame temperature of 1000 °C: **(d_1_-d_3_)** PSC-EM; **(e_1_-e_3_)** PSC-EE; **(f_1_-f_3_)** PSC-MM; **(g_1_-g_3_)** PSC-ME.

**Table S1. Formulation details and mass changes during SAEs synthesis.**

| SAE | Amino silane (mol) | Epoxy silane (mol) | Distillate mass (g) | Distillate composition (MeOH / EtOH mol%) | Evolved alcohol (mol) | Reaction extent (%) | Apparent viscosity (Pa·s) |
| --- | --- | --- | --- | --- | --- | --- | --- |
| SAE-MM | 0.5 | 1 | 29.85 | 100/0 | 0.932 | 93.2 | 2.05 |
| SAE-EM | 0.5 | 1 | 33.08 | 57/43 | 0.869 | 86.9 | 4.76 |
| SAE-ME | 0.5 | 1 | 30.49 | 62/38 | 0.816 | 81.6 | 0.65 |
| SAE-EE | 0.5 | 1 | 32.94 | 0/100 | 0.715 | 71.5 | 0.46 |

**Table S2. Amount of Deionized Water for PSA Preparation with Various SAEs**

| SAE | Deionized Water  (g) |
| --- | --- |
| SAE-EM | 0.82 |
| SAE-EE | 0.73 |
| SAE-MM | 0.87 |
| SAE-ME | 0.77 |

**Table S3. Typical physical properties of the aerogels**

| Sample | Bulk density  (g·cm^-3^) | Volume shrinkage (%) | Mean pore diameter *^a^* (nm) | S_BET_ *^b^*  (m^2^·g^-1^) | Pore volume  (cm^3^·g^-1^) | λ *^c^*  (W·m^-1^·K^-1^) |
| --- | --- | --- | --- | --- | --- | --- |
| PSA-EM | 0.33 | 2 | 12.1 | 435 | 1.59 | 0.032 |
| PSA-EE | 0.32 | 2 | 10.9 | 485 | 1.45 | 0.031 |
| PSA-MM | 0.35 | 7 | 9.4 | 536 | 1.42 | 0.034 |
| PSA-ME | 0.33 | 3 | 9.8 | 537 | 1.43 | 0.032 |

*^a^*Mean pore diameter obtained from nitrogen adsorption branch via the Barrett−Joyner−Halenda method. *^b^*Brunauer−Emmett−Teller SSA obtained from nitrogen adsorption measurement. *^c^*Thermal conductivity at room temperature and ambient pressure.

**Table S4. GC/MS (TG-FTIR-GCMS) analysis of the volatile pyrolysis products derived from PSA-EM from 500-800°C**

| No. | Product | Retention Time  (min) | Molecular formula | Relative Content (%) |
| --- | --- | --- | --- | --- |
| 1 | 3-Methylpentanal | 2.17 | C_6_H_12_O | 6.18 |
| 2 | Methyl Methacrylate | 2.41 | C_5_H_8_O_2_ | 28.73 |
| 3 | 2-Propenoic Acid, Butyl Ester | 4.19 | C_7_H_12_O_2_ | 5.36 |
| 4 | n-Butyl Methacrylate | 5.80 | C_8_H_14_O_2_ | 5.65 |
| 5 | 2-Quinazolinamine, 3,4-Dihydro-N,3-Diphenyl | 11.58 | C_26_H_20_N_4_ | 2.54 |
| 6 | Octamethyl-  silsesquioxane | 14.49/14.89 | C_8_H_24_O_12_Si_8_ | 10.80 |
| 7 | 1-Diphenyl(tert-butyl)silyloxy-4-nitrobenzene | 17.54/17.71 | C_22_H_23_O_3_NSi | 1.32 |
| 8 | Hexadecanoic Acid, Methyl Ester | 20.33 | C_17_H_34_O_2_ | 4.37 |
| 9 | Methyl Stearate | 22.69 | C_19_H_38_O_2_ | 6.98 |
| 10 | Others | \ | \ | 28.07 |

**Table S5. GC/MS (TG-FTIR-GCMS) analysis of the volatile pyrolysis products derived from PSA-MM from 500-800°C**

| No. | Product | Retention Time  (min) | Molecular formula | Relative Content (%) |
| --- | --- | --- | --- | --- |
| 1 | 2(3H)-Furanone, Dihydro-3-Methyl- | 2.16 | C_5_H_8_O_2_ | 6.51 |
| 2 | Methyl Methacrylate | 2.40 | C_5_H_8_O_2_ | 21.82 |
| 3 | 2-Propenoic Acid, Butyl Ester | 4.39 | C_7_H_12_O_2_ | 4.13 |
| 4 | n-Butyl Methacrylate | 5.77 | C_8_H_14_O_2_ | 5.55 |
| 5 | 2-Quinazolinamine, 3,4-Dihydro-N,3-Diphenyl | 11.55 | C_26_H_20_N_4_ | 3.53 |
| 6 | Octamethyl-  silsesquioxane | 14.47/14.87 | C_8_H_24_O_12_Si_8_ | 13.43 |
| 7 | 1-Diphenyl(tert-butyl)silyloxy-4-nitrobenzene | 17.54/17.75 | C_22_H_23_O_3_NSi | 3.89 |
| 8 | Hexadecanoic Acid, Methyl Ester | 20.33 | C_17_H_34_O_2_ | 2.11 |
| 9 | Methyl Stearate | 22.69 | C_19_H_38_O_2_ | 3.63 |
| 10 | Others | \ | \ | 35.40 |

**Table S6. GC/MS (TG-FTIR-GCMS) analysis of the volatile pyrolysis products derived from PSA-ME from 500-800°C**

| No. | Product | Retention Time  (min) | Molecular formula | Relative Content (%) |
| --- | --- | --- | --- | --- |
| 1 | 2(3H)-Furanone, Dihydro-3-Methyl- | 2.14 | C_5_H_8_O_2_ | 5.05 |
| 2 | Methyl Methacrylate | 2.36 | C_5_H_8_O_2_ | 7.95 |
| 3 | 2-Propenoic Acid, Butyl Ester | 4.39 | C_7_H_12_O_2_ | 1.67 |
| 4 | n-Butyl Methacrylate | 5.76 | C_8_H_14_O_2_ | 3.57 |
| 5 | 2-Quinazolinamine, 3,4-Dihydro-N,3-Diphenyl | 11.54 | C_26_H_20_N_4_ | 6.98 |
| 6 | Octamethyl-  silsesquioxane | 14.46/14.87 | C_8_H_24_O_12_Si_8_ | 23.42 |
| 7 | 1-Diphenyl(tert-butyl)silyloxy-4-nitrobenzene | 17.52/17.74 | C_22_H_23_O_3_NSi | 7.72 |
| 8 | Hexadecanoic Acid, Methyl Ester | 20.32 | C_17_H_34_O_2_ | 1.34 |
| 9 | Methyl Stearate | 22.67 | C_19_H_38_O_2_ | 1.64 |
| 10 | Others | \ | \ | 40.66 |

**Table S7. GC/MS (TG-FTIR-GCMS) analysis of the volatile pyrolysis products derived from PSA-EE from 500-800°C**

| No. | Product | Retention Time  (min) | Molecular formula | Relative Content (%) |
| --- | --- | --- | --- | --- |
| 1 | 2(3H)-Furanone, Dihydro-3-Methyl- | 2.17 | C_5_H_8_O_2_ | 4.58 |
| 2 | Methyl Methacrylate | 2.38 | C_5_H_8_O_2_ | 15.30 |
| 3 | 2-Propenoic Acid, Butyl Ester | 4.39 | C_7_H_12_O_2_ | 2.71 |
| 4 | n-Butyl Methacrylate | 5.77 | C_8_H_14_O_2_ | 3.99 |
| 5 | 2-Quinazolinamine, 3,4-Dihydro-N,3-Diphenyl | 11.55 | C_26_H_20_N_4_ | 5.49 |
| 6 | Octamethyl-  silsesquioxane | 14.47/14.87 | C_8_H_24_O_12_Si_8_ | 20.27 |
| 7 | 1-Diphenyl(tert-butyl)silyloxy-4-nitrobenzene | 17.52/17.74 | C_22_H_23_O_3_NSi | 3.07 |
| 8 | Hexadecanoic Acid, Methyl Ester | 20.32 | C_17_H_34_O_2_ | 1.87 |
| 9 | Methyl Stearate | 22.67 | C_19_H_38_O_2_ | 4.44 |
| 10 | Others | \ | \ | 38.28 |

**Table. S8 Basic information of different PSAs molecular models.**

| Simulation model | Atoms | Formula | Density  (g·cm^-3^) | Volume  (Å^3^) |
| --- | --- | --- | --- | --- |
| PSA-EM | 11316 | C_2124_H_6048_O_2172_N_60_Si_912_ | 0.3 | 80*80*80 |
| PSA-MM | 11238 | C_2142_H_6012_O_2154_N_60_Si_870_ | 0.3 | 80*80*80 |
| PSA-ME | 11728 | C_2192_H_6312_O_2232_N_56_Si_936_ | 0.3 | 80*80*80 |
| PSA-EE | 12024 | C_2344_H_6536_O_2184_N_56_Si_904_ | 0.3 | 81*81*81 |

**Table S9. Py-GC/MS analysis of the volatile pyrolysis products relative content (%)**

| Sample | C_2_H_4_ | Hexamethylcyclotrisiloxane  (D3) | Octamethylcyclotetrasiloxane  (D4) | Decamethylcyclopentasiloxane (D5) | Dodecamethylcyclohexasiloxane  (D6) | Octamethylsilsesquioxane  (POSS) | Other |
| --- | --- | --- | --- | --- | --- | --- | --- |
| PSC-EM | 16.20 | 15.04 | 3.62 | 2.83 | 2.69 | 36.69 | 22.93 |
| PSC-EE | 32.93 | 14.51 | 3.78 | 2.65 | 1.85 | 30.24 | 14.04 |
| PSC-MM | 25.30 | 15.83 | 3.45 | 2.54 | 2.34 | 33.02 | 17.52 |
| PSC-ME | 27.20 | 13.46 | 4.05 | 2.43 | 2.39 | 32.21 | 18.26 |

**Reference**

[1] H. Cai, B. Niu, Z. Qian, T. Li, P. Wang, L. Li, Y. Cao, Y. Zhang, D. Long, *Composites Science and Technology* **2024**, *245*, 110325.

[2] H. H. Horowitz, Gershon. Metzger, *Anal. Chem.* **1963**, *35*, 1464.

[3] F. Zheng, Z. Ren, B. Xu, K. Wan, J. Cai, J. Yang, T. Zhang, P. Wang, B. Niu, Y. Zhang, D. Long, *Journal of Analytical and Applied Pyrolysis* **2021**, *157*, 105222.

[4] Y. Ma, Y. Yang, C. Lu, X. Wen, X. Liu, K. Lu, S. Wu, Q. Liu, *Composites Science and Technology* **2018**, *167*, 53.

[5] S. Yan, D. Xia, X. Zhang, B. Jiang, *Waste Management* **2019**, *93*, 83.

[6] M. Wu, T. Wu, Y. Luo, M. Xu, B. Niu, Y. Xing, Y. Zhang, D. Long, *J. Anal. Appl. Pyrolysis* **2025**, *188*, 107038.

[7] H. Liu, G. Zhu, C. Zhang, *Composites Part B: Engineering* **2020**, *190*, 107901.

[8] Z. Yu, N. Yang, V. Apostolopoulou‐Kalkavoura, B. Qin, Z. Ma, W. Xing, C. Qiao, L. Bergström, M. Antonietti, S. Yu, *Angew. Chem. - Int. Ed.* **2018**, *57*, 4538.

[9] Y. Luo, A. Yan, H. Tian, B. Niu, Y. Zhang, H. Wang, D. Long, *J. Mater. Chem.A* **2024**, *12*, 4684.

[10] A. Yan, Y. Luo, H. Tian, H. Pan, Y. Cao, B. Niu, Y. Zhang, D. Long, *J. Colloid Interface Sci.* **2024**, *663*, 665.

[11] A. Borba, J. P. Vareda, L. Durães, A. Portugal, P. N. Simões, *New J. Chem.* **2017**, *41*, 6742.

[12] S. Zhao, W. J. Malfait, E. Jeong, B. Fischer, Y. Zhang, H. Xu, E. Angelica, W. M. Risen, J. W. Suggs, M. M. Koebel, *ACS Sustainable Chem. Eng.* **2016**, *4*, 5674.

[13] J. Ren, X. Huang, J. Shi, W. Wang, J. Li, Y. Zhang, H. Chen, R. Han, G. Chen, Q. Li, Z. Zhou, *J. Colloid Interface Sci.* **2022**, *623*, 1101.
